# Visible-light degradation of ciprofloxacin by hierarchical Ag_3_PO_4_/CuInS_2_ heterostructures: interfacial charge transfer, transformation pathways, and ecological risk context

**DOI:** 10.1039/d6ra09598d

**Published:** 2026-11-12

**Authors:** Adewumi O. Oluwole, Nokwazi C. Mahlangu, Phephile Ngwenya, Shepherd M. Tichapondwa, Michael O. Daramola, Samuel A. Iwarere

**Affiliations:** a Department of Chemical Engineering, Faculty of Engineering, Built Environment and Information Technology, University of Pretoria Hatfield Pretoria 0002 South Africa omayor8@gmail.com samuel.iwarere@up.ac.za +27-12-420-3092

## Abstract

Ciprofloxacin persists in aquatic environments and may contribute to antimicrobial resistance, creating demand for efficient, low-energy treatment technologies. In this study, a hierarchical flower-like Ag_3_PO_4_/CuInS_2_ heterostructure was synthesized through a combined solvothermal–hydrothermal route and evaluated for visible-light-driven ciprofloxacin degradation. XRD, FTIR, Raman, XPS and elemental mapping confirmed the coexistence and interfacial coupling of cubic Ag_3_PO_4_ and chalcopyrite CuInS_2_. The heterostructure consisted of faceted Ag_3_PO_4_ particles integrated with flower-like CuInS_2_ aggregates and exhibited a surface area of 39.22 m^2^ g^−1^, average pore diameter of 47.70 nm and band gap of 1.92 eV. Photoluminescence quenching and a reduced impedance arc indicated improved interfacial charge migration and suppressed electron–hole recombination. Under visible light, the composite achieved 96.13% ciprofloxacin degradation within 60 min, compared with 44.30% for Ag_3_PO_4_ and 58.87% for CuInS_2_, with a rate constant of 0.03561 min^−1^, 5.3 and 3.76 times higher, respectively. Maximum degradation occurred near pH 6.6, while excessive catalyst loading and higher ciprofloxacin concentration reduced efficiency. Radical-quenching experiments showed that photogenerated holes and hydroxyl radicals were the dominant oxidative species, with superoxide making a secondary contribution. A direct Z-scheme-like pathway was proposed, retaining strongly oxidizing holes in the Ag_3_PO_4_ valence band and reducing electrons in the CuInS_2_ conduction band. LC-MS indicated piperazine oxidation, *N*-dealkylation, ring opening, defluorination, decarboxylation and quinolone-ring cleavage. The heterostructure retained 89.45% degradation efficiency after four cycles, indicating promising reusability. These findings demonstrate enhanced charge separation, rapid antibiotic transformation, improved photocatalytic performance and promising visible-light treatment potential of antibiotic-contaminated water.

## Abstract 2

Ciprofloxacin persists in aquatic environments and may contribute to antimicrobial resistance, creating demand for efficient, low-energy treatment technologies.

## Graphical abstract

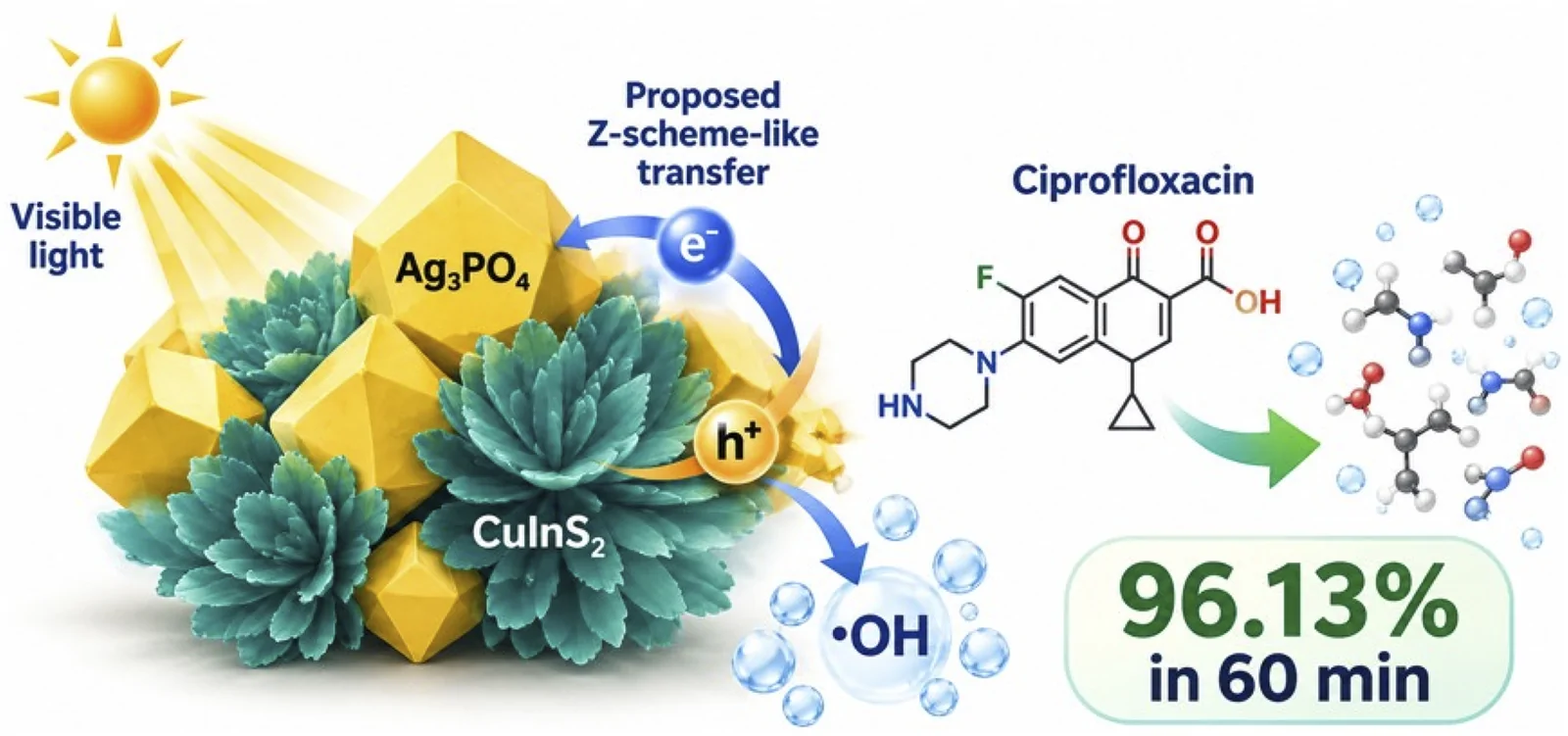

## 1 Introduction

The continuous discharge of antibiotics into aquatic environments has become a serious environmental and public health concern due to their persistence, biological activity, and potential contribution to antimicrobial resistance. Antibiotics are widely used in human healthcare, veterinary medicine, aquaculture, livestock production, and related industrial sectors, but they are often incompletely metabolized and subsequently released into wastewater, surface water, groundwater, sludge, and soil systems.^1–3^ Even at trace concentrations, their continuous input into the environment can exert selective pressure on microorganisms, promote resistant bacterial strains, and disrupt aquatic ecosystems. This concern is particularly important for fluoroquinolone antibiotics, which are known for their chemical stability, broad-spectrum antimicrobial activity, and poor biodegradability.^4,5^ Recent studies continue to identify ciprofloxacin as an environmentally persistent antibiotic of concern, with risks linked to aquatic toxicity and the promotion of antibiotic resistance in water systems. Ciprofloxacin (CIP) is among the most frequently used fluoroquinolone antibiotics for the treatment of bacterial infections in humans and animals.^6^ The persistence of CIP in water is concerning because it can inhibit the growth of aquatic organisms, alter microbial communities, and contribute to the development and spread of antibiotic-resistant bacteria with Fig. S1 providing additional information about the occurrence of ciprofloxacin in global surface water matrices.^7,8^ Therefore, the development of efficient, stable, and sustainable technologies for CIP removal is an important research priority in environmental chemical engineering.

Several treatment technologies, including adsorption, membrane filtration, ion exchange, electrochemical oxidation, anaerobic membrane bioreactors, and advanced oxidation processes, have been investigated for antibiotic removal.^9–11^ Although these methods can reduce antibiotic concentrations, they often suffer from limitations such as high operational cost, incomplete mineralization, secondary waste generation, membrane fouling, chemical consumption, and difficulty in catalyst or adsorbent regeneration. In this context, heterogeneous photocatalysis has attracted considerable attention because it can utilize light energy to generate reactive oxygen species capable of degrading persistent organic pollutants into less harmful intermediates and, ideally, mineralized products such as CO_2_ and H_2_O.^12^ Among visible-light-responsive photocatalysts, silver orthophosphate (Ag_3_PO_4_) has gained significant attention due to its narrow band gap, strong visible-light absorption, and excellent photooxidative ability.^13^ Since its discovery as a highly active visible light photocatalyst, Ag_3_PO_4_ has been widely investigated for the degradation of organic contaminants, including dyes, pharmaceuticals, pesticides, and other emerging pollutants. Its strong valence-band oxidation potential enables the generation of highly reactive oxidative species, making it particularly suitable for the degradation of recalcitrant pollutants such as ciprofloxacin.^14^ However, the practical application of Ag_3_PO_4_ remains limited by severe photocorrosion, instability during repeated photocatalytic cycles, poor dispersibility in aqueous systems, and the possible reduction of Ag^+^ to metallic Ag under irradiation.^15,16^ These limitations reduce its long-term photocatalytic efficiency and hinder its scale-up for wastewater treatment applications.

To overcome these drawbacks, coupling Ag_3_PO_4_ with a suitable semiconductor to construct a heterojunction is a promising strategy. Semiconductor coupling can improve visible-light harvesting, promote interfacial charge separation, suppress electron–hole recombination, and reduce the photocorrosion of Ag_3_PO_4_ by facilitating the migration of photogenerated electrons away from the Ag_3_PO_4_ surface. Copper indium sulfide (CuInS_2_) is an attractive partner for this purpose because of its narrow band gap, strong visible-light absorption, relatively low toxicity, tunable optical properties, and favorable photocatalytic characteristics.^17,18^ CuInS_2_ has been investigated for solar energy conversion, light-emitting devices, photocatalysis, and water purification, and its band structure makes it suitable for constructing charge-transfer heterojunctions with visible-light-active semiconductors.^19,20^ The construction of an Ag_3_PO_4_/CuInS_2_ heterostructure is therefore expected to address the main limitations of pristine Ag_3_PO_4_ while enhancing photocatalytic degradation performance under visible light. In such a composite system, Ag_3_PO_4_ can provide strong oxidative ability, while CuInS_2_ can act as a visible-light absorber and charge-transfer mediator. The intimate interfacial contact between both semiconductors may facilitate directional separation of photogenerated electrons and holes, thereby improving the lifetime of charge carriers and increasing the formation of reactive oxidative species. This charge-transfer pathway is also expected to reduce the accumulation of electrons on Ag_3_PO_4_, thereby mitigating Ag^+^ reduction and improving photocatalyst stability. Based on the supplied study rationale, the proposed Ag_3_PO_4_/CuInS_2_ nanocomposite is designed specifically for sustainable photocatalytic degradation of ciprofloxacin in simulated water under visible-light irradiation.

In this study, a hierarchical flower-Ag_3_PO_4_/CuInS_2_ heterostructure was prepared through a combined solvothermal–hydrothermal route for the visible-light-driven degradation of ciprofloxacin. The structural, morphological, surface, optical and electronic properties of the individual semiconductors and heterostructure were systematically investigated. Particular attention was given to determining how interfacial coupling influences visible-light absorption, band alignment, charge separation and charge-transfer resistance. The photocatalytic performance was evaluated through degradation kinetics, operational-parameter studies, reactive-species trapping, mineralization and recyclability tests. This integrated approach was intended to clarify the structure–property–activity relationship responsible for the enhanced activity and stability of the Ag_3_PO_4_/CuInS_2_ heterostructure.

## 2 Experimental

### 2.1 Materials

Ammonium dihydrogen phosphate (NH_4_H_2_PO_4_), ethanol (C_2_H_6_O), methanol (CH_4_O), silver nitrate (AgNO_3_), copper nitrate (Cu(NO_3_)_2_·3H_2_O), indium(iii) chloride (InCl_3_), thioacetamide (C_2_H_5_NS), acetonitrile (CH_3_CN), ciprofloxacin (C_17_H_18_FN_3_O_3_), dimethylformamide (DMF), sodium hydroxide (NaOH), *p*-benzoquinone (*p*-BQ), isopropyl alcohol (IPA, 99.9%), triethanolamine (TEA), sodium hydroxide (NaOH) were acquired from Sigma-Aldrich, South Africa. All chemicals are of fine analytical grade; hence, they were used without further purification.

### 2.2 Synthesis procedure

#### 2.2.1 Synthesis of CuInS_2_ composites

Using a solvothermal technique, stoichiometric quantities of copper nitrate (1 mmol), indium chloride (1 mmol), and thioacetamide (2 mmol) were dissolved separately in 20 mL of DMF before being combined and agitated for one hour to form a homogeneous mixture. After that, the homogeneous mixture was put in an autoclave and treated for 12 hours at 180 °C. Following the reaction, the obtained precipitate was carefully collected *via* centrifugation, cleaned with deionized water, ethanol and allowed to dry at 70 °C for the entire night.

#### 2.2.2 Preparation of Ag_3_PO_4_/CuInS_2_ heterostructures

The hydrothermal procedure was used in producing the Ag_3_PO_4_/CuInS_2_ heterostructure composites. To achieve a homogeneous suspension, 50 mg of the as-synthesized CuInS_2_ composite sample was added to 75 mL of distilled water and sonicated for 10 min. Under continuous stirring, 3 mmol of silver nitrate (AgNO_3_) was then added to the solution. To maintain the stoichiometric molar ratio of AgNO_3_ and NH_4_H_2_PO_4_ (3 : 1), 1 mmol of ammonium dihydrogen phosphate NH_4_H_2_PO_4_, dissolved in 25 mL of distilled water was then added dropwise to the solution. For one hour, the resultant suspension was kept constantly agitated. After the resulting precipitate was processed for 24 hours at 100 degrees Celsius in an autoclave oven, the synthesized material was cleaned with ethanol and distilled water and dried for 12 hours at 60 degrees Celsius to obtain the Ag_3_PO_4_/CuInS_2_ heterostructure composites in mole ratio of 5 : 1. For comparison, the same conditions were used to prepare pure Ag_3_PO_4_ without the addition of CuInS_2_.

### 2.3 Characterization instrumentation

The characterization details are included in the SI as Text S2.

### 2.4 Photocatalytic activity test

The photocatalytic degradation of ciprofloxacin solution was performed to evaluate the photocatalytic activity of the Ag_3_PO_4_, CuInS_2_ and Ag_3_PO_4_/CuInS_2_ heterostructure composites. The tests were conducted under simulated visible-light irradiation using the reactor setup with 72 W fluorescent lamps positioned approximately 10 cm from the reaction vessel with light intensity at approximately 142.86 W m^−2^. A 50 mg of the synthesized composites was added to 500 mL of CIP solution with a concentration of 7 ppm. Before conducting the CIP degradation test, the solution was left in the dark for 30 min with constant stirring to achieve CIP adsorption/desorption equilibrium. After stirring in the dark for 30 min, the mixture was irradiated with the simulated visible light while 4 mL of the sample solution were taken at every 10 min, centrifuged, and filtered using a 0.45 µm membrane filter to remove residual catalyst particles. The filtrate was then analyzed using a UV-vis spectrophotometer (Biochrom, Cambridge, UK) by monitoring the absorbance at 276 nm to determine the pollutant concentration during degradation. The degradation efficiency (%) was calculated based on eqn (1), while the degradation rate was calculated using the pseudo-first-order model as shown in eqn (2).1
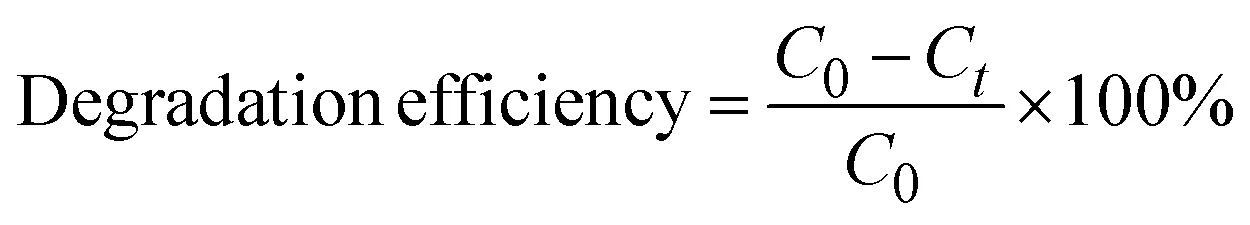
2
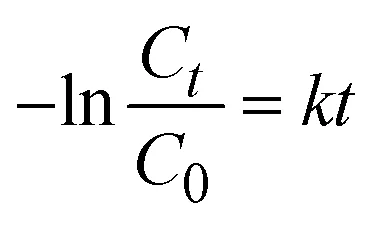
where *k* (min^−1^) denotes the apparent rate constant, *C*_0_ is the initial concentration of CIP, and *C*_*t*_ (mg L^−1^) represents the CIP concentration at a given irradiation time.

The contributions of different reactive species to ciprofloxacin (CIP) degradation were evaluated through scavenger experiments. 30 mg of 1,4-benzoquinone (BQ) was used to quench superoxide radicals (˙O_2_^−^), 20 µL of triethanolamine (TEOA) was employed as a scavenger for photogenerated holes (h^+^), and 20 µL isopropanol (IPA) was used to inhibit hydroxyl radicals (˙OH). The quantities of the scavengers were selected to provide sufficient molar concentrations for effective competition with ciprofloxacin for the corresponding reactive species without excessively altering the composition of the photocatalytic suspension which is sufficient for comparative quenching experiments while limiting excessive dilution and possible interference with light penetration or catalyst dispersion. The cyclic reusability experiment was conducted under optimized photocatalytic conditions. 50 mg of the Ag_3_PO_4_/CuInS_2_ composite was dispersed in the 500 mL CIP solution at its natural initial pH of approximately 6.6 and initial concentration of 7 mg L^−1^. After each cycle, the photocatalyst was recovered by centrifugation, washed several times with deionized water to remove residual CIP and adsorbed degradation intermediates, and dried before reuse. The recovered catalyst was subsequently introduced into a freshly prepared CIP solution, and this procedure was repeated for four consecutive cycles. No additional catalyst was added between cycles, allowing the observed change in degradation efficiency to reflect the reusability and operational stability of the composite. A Shimadzu TOC-V analyzer was used to quantitatively evaluate the mineralization of ciprofloxacin by measuring the total organic carbon (TOC) concentrations before and after the photocatalytic reaction. The TOC removal efficiency was calculated using equation proposed by ref. 21. All experiments were conducted in triplicate for reproducibility.

## 3 Results and discussion

### 3.1 Structural phase and functional group analysis

The structural and functional properties of the fabricated semiconductor materials and their heterostructure were systematically investigated using X-ray diffraction (XRD), Fourier transform infrared (FTIR) and Raman spectroscopy. The pristine Ag_3_PO_4_ sample exhibits several sharp and intense diffraction peaks across the 2*θ* range of 10–80°, indicating its highly crystalline nature. As shown in Fig. 1a, the major diffraction reflections occur at approximately 24.2°, 34.7°, 38.9°, 42.7°, 49.8°, 56.0°, 62.0°, 64.8°, 67.6°, and 73.1° indexed to the characteristic planes of crystalline Ag_3_PO_4_, including the (110), (200), (210), (211), (220), (310), (222), (320), (321), and (400) crystal planes of cubic Ag_3_PO_4_, which is in good agreement with JCPDS card No. 00-006-0505.^22^ The CuInS_2_ pattern displays broader and less intense diffraction features, approximately at 2*θ* = 32.6°, 37.2°, 56.3°, 65.1°, and a weak shoulder around 68–70° which can be indexed to the (112), (004), (204)/(220), (116)/(312), and (008)/(400) planes of tetragonal chalcopyrite CuInS_2_, respectively, in agreement with JCPDS No. 27-0159. The broad nature of the diffraction peaks suggests the formation of nanocrystalline CuInS_2_ with relatively small crystallite size and/or lattice defects.^23,24^ The XRD patterns show the phase evolution and successful coupling of Ag_3_PO_4_ with CuInS_2_ in the Ag_3_PO_4_/CuInS_2_ heterostructure as the diffraction profile retains the major characteristic peaks of Ag_3_PO_4_, while weak and broadened CuInS_2_-related features are also observed, confirming the coexistence of both semiconductors in the hybrid material. The absence of obvious additional impurity peaks suggests that no major secondary crystalline phases were formed during composite fabrication. Moreover, the retention of the Ag_3_PO_4_ diffraction pattern without pronounced peak displacement indicates that the formation of Ag_3_PO_4_/CuInS_2_ occurs mainly through interfacial coupling rather than lattice substitution.^25,26^ This intimate heterostructure formation is beneficial for photocatalytic application because the crystalline Ag_3_PO_4_ framework can provide strong photooxidative sites, while the incorporated CuInS_2_ phase may enhance visible-light absorption and facilitate interfacial charge separation, thereby improving the photocatalytic degradation performance of the composite.^27^ The average crystallite size of the synthesized materials was estimated using the Debye–Scherrer equation from the major diffraction peak of the composite as provided in Text S3. The crystallite sizes were estimated to be approximately 52, 16, and 38 nm for Ag_3_PO_4_, CuInS_2_ and Ag_3_PO_4_/CuInS_2_, respectively. The reduction in crystallite size in the composite compared with pristine Ag_3_PO_4_ may be attributed to the interfacial incorporation of CuInS_2_, which can restrict crystal growth and promote heterojunction formation.^28^

**Fig. 1:**
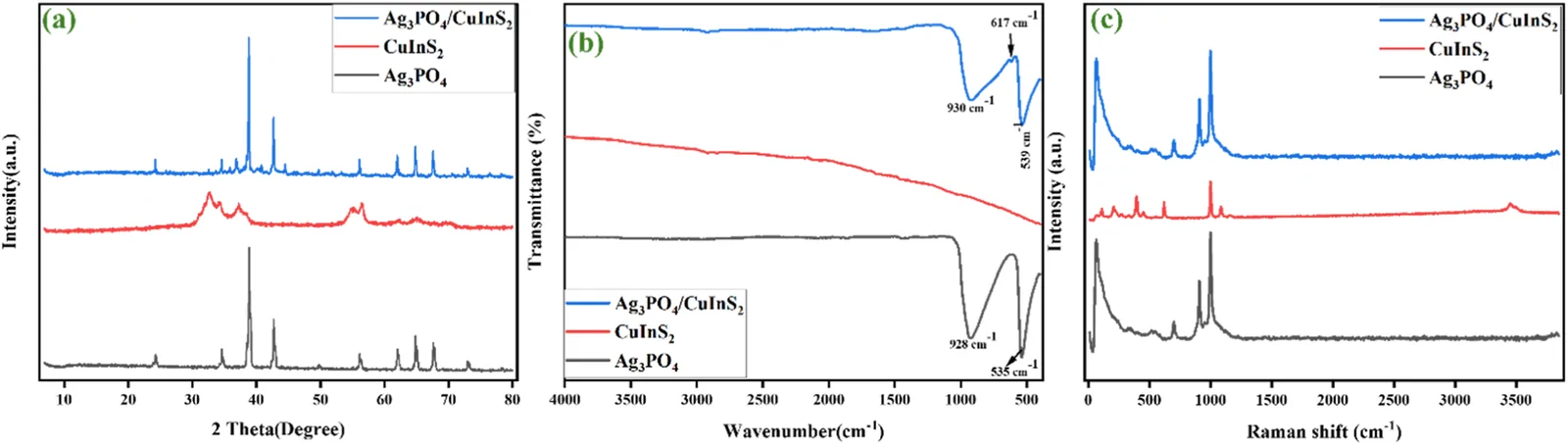
(a) X-ray diffractograms, (b) FTIR spectra and (c) Raman spectra of Ag_3_PO_4_, CuInS_2_ and Ag_3_PO_4_/CuInS_2_ heterostructure composites.

The FTIR spectra further support the formation of the Ag_3_PO_4_/CuInS_2_ heterojunction and the preservation of the main vibrational features of Ag_3_PO_4_ after coupling with CuInS_2_. For pristine Ag_3_PO_4_, the strong absorption band at 928 cm^−1^ can be assigned to the stretching vibration of the P–O bonds in the PO_4_^3−^ tetrahedral unit, while the band at 535 cm^−1^ is associated with the bending vibration of the PO_4_^3−^ group and Ag–O related lattice vibration (Fig. 1b). Similar FTIR bands have been reported for Ag_3_PO_4_, where absorption around 925 cm^−1^ was attributed to P–O stretching and the band around 530 cm^−1^ to PO_4_^3−^ bending vibration.^29^ In the Ag_3_PO_4_/CuInS_2_ composite, these characteristic bands are retained but slightly shifted to 930 cm^−1^ and 539 cm^−1^, suggesting that the Ag_3_PO_4_ phosphate framework remains intact while experiencing interfacial interaction with CuInS_2_. The additional band observed at 617 cm^−1^ may be related to low-wavenumber metal–sulfide lattice vibration or interfacial bonding between Ag_3_PO_4_ and CuInS_2_. The slight shift of the Ag_3_PO_4_ bands in the composite is significant because shifts in FTIR band positions after coupling two semiconductor phases have been linked to changes in bond environment and strong interfacial interaction in heterostructure composites. Therefore, the coexistence of Ag_3_PO_4_ vibrational features with the shifted bands in the composite indicates successful heterojunction formation between Ag_3_PO_4_ and CuInS_2_.

Raman analysis confirmed the coexistence and interfacial coupling of Ag_3_PO_4_ and CuInS_2_ in the heterostructure. Pure Ag_3_PO_4_ exhibited characteristic bands at approximately 100–230 cm^−1^, attributed to translational and rotational vibrations of the [PO_4_]^3−^ units, together with phosphate bending and O–P–O stretching vibrations near 550–560, 910, and 990–1000 cm^−1^.^30^ CuInS_2_ showed bands at about 110, 205, 396, 451, 618, 1000, 1085, and 3443 cm^−1^, with the low-wavenumber features assigned to Cu–In–S lattice and metal–sulfur vibrations, while the bands at 396 and 451 cm^−1^ may arise from defect-activated Cu–S modes or surface sulfide species. The weak or absent characteristic A_1_ mode near 290–294 cm^−1^ suggests lattice disorder, low crystallinity, or cation disorder,^31,32^ whereas the higher-wavenumber bands are likely associated with surface oxyanion residues and adsorbed hydroxyl species. In the Ag_3_PO_4_/CuInS_2_ composite as shown in Fig. 1c, the retention of the main Ag_3_PO_4_ phosphate bands alongside CuInS_2_-related low-wavenumber features confirms the presence of both phases, while slight band broadening and intensity changes indicate interfacial interactions, local lattice distortion, defect formation, and possible electronic coupling at the heterojunction interface.

### 3.2 Microstructural analysis

The morphological features of the synthesized materials were evaluated and as shown in Fig. 2a, the SEM image of pure Ag_3_PO_4_ shows well-defined faceted, plate-like and irregular polyhedral particles with smooth surfaces and sharp edges with clear geometric features indicate good crystallinity, consistent with the sharp peaks observed in the XRD pattern. The SEM image of CuInS_2_ shows a highly rough and porous morphology made up of interconnected thin flakes arranged into flower-like aggregates. The curled and overlapping sheets create open pores and surface voids, indicating a hierarchical structure with potentially high surface accessibility. The SEM image of the Ag_3_PO_4_/CuInS_2_ composite as shown in Fig. 2c, shows a clear hybrid morphology, where faceted plate-like Ag_3_PO_4_ particles are closely integrated with rough, flower-like CuInS_2_ aggregates. The coexistence of these two morphologies suggests the successful coupling of Ag_3_PO_4_ and CuInS_2_ within the composite. The CuInS_2_ appears as rounded, textured microspherical clusters distributed around or on the Ag_3_PO_4_ plates, creating intimate interfacial contact between both phases. This heterostructure arrangement is beneficial for photocatalysis because it can provide more active surface sites, improve pollutant adsorption, and facilitate interfacial charge transfer between Ag_3_PO_4_ and CuInS_2_.^33,34^ Overall, the SEM image supports the formation of a hierarchical flower-like Ag_3_PO_4_/CuInS_2_ heterojunction composite, which may enhance visible-light-driven photocatalytic performance.

**Fig. 2:**
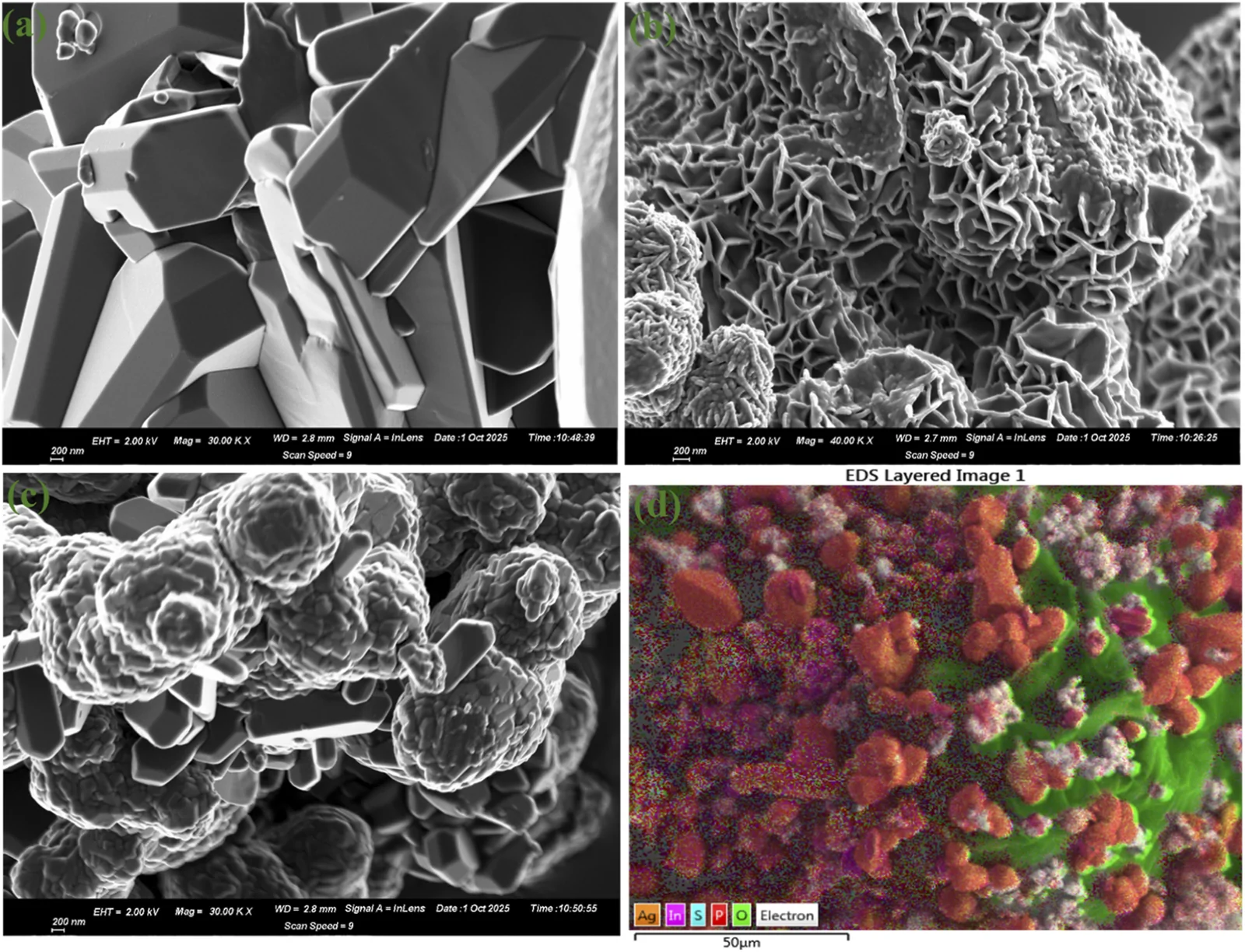
SEM images of (a) Ag_3_PO_4_, (b) CuInS_2_, (c) Ag_3_PO_4_/CuInS_2_ and (d) EDX mapping of Ag_3_PO_4_/CuInS_2_ composites.

The EDS elemental mapping and spectrum of the Ag_3_PO_4_/CuInS_2_ heterojunction composite as shown in Fig. 3, confirms the successful incorporation and distribution of Ag, P, O, Cu, In, and S within the synthesized material. The strong signals of Ag, P, and O indicate the dominant presence of the Ag_3_PO_4_ phase, while the detection of Cu, In, and S confirms the introduction of CuInS_2_ into the composite. The partial overlap of these elements across the mapped region suggests intimate contact between Ag_3_PO_4_ and CuInS_2_ supporting heterojunction formation. The EDS spectrum further shows Ag as the major component, followed by O and P, while the lower Cu, In, and S contents reflect the relatively smaller proportion of CuInS_2_ in the composite. Overall, the elemental mapping verifies the coexistence of both semiconductor phases and supports the formation of a well-integrated Ag_3_PO_4_/CuInS_2_ heterojunction, which can promote interfacial charge transfer and enhance photocatalytic performance.

**Fig. 3:**
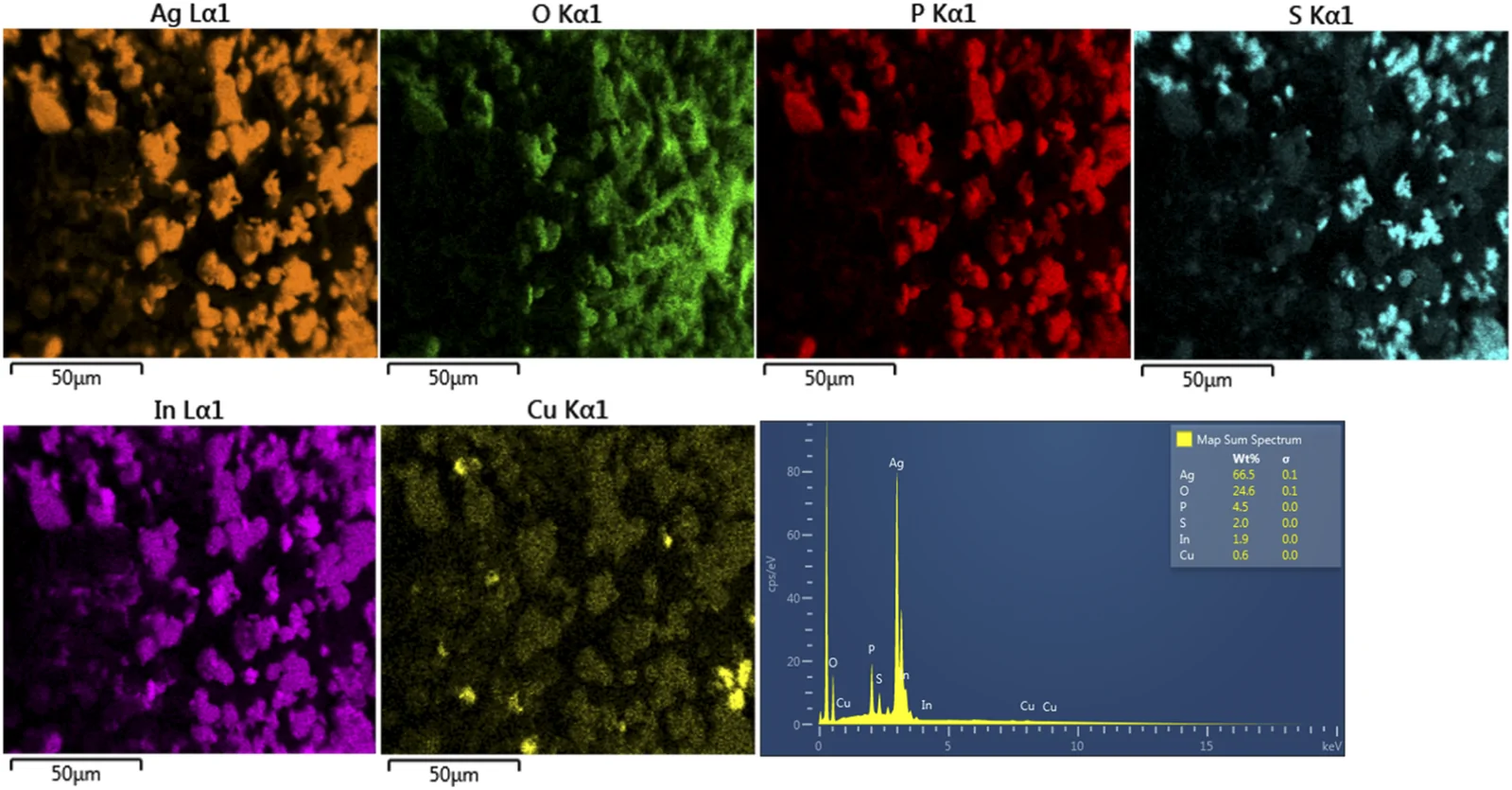
EDX images of Ag_3_PO_4_/CuInS_2_ heterostructure composites.

### 3.3 Surface elemental analysis

The electronic structure and chemical environment at the boundary between Ag_3_PO_4_, CuInS_2_ the Ag_3_PO_4_/CuInS_2_ heterostructure composites are further examined *via* X-ray photoelectron spectroscopy (XPS). As shown in Fig. 4a, the XPS survey spectra confirm the successful formation of the Ag_3_PO_4_/CuInS_2_ heterojunction, as the composite exhibits the characteristic elemental signals of both Ag_3_PO_4_ and CuInS_2_, including Ag 3d, P 2p, O 1s, Cu 2p, In 3d and S 2p, while the absence of obvious impurity peaks indicates good compositional purity, and the coexistence of these elements in the composite demonstrates effective coupling between the two semiconductors, which is essential for interfacial charge transfer and improved photocatalytic performance. The Ag 3d XPS scan (Fig. 4b) shows two well-resolved spin–orbit peaks for pristine Ag_3_PO_4_ at 367.8 and 373.6 eV, corresponding to Ag 3d_5/2_ and Ag 3d_3/2_ of Ag^+^ in Ag_3_PO_4_, while the Ag_3_PO_4_/CuInS_2_ composite exhibits the same doublet slightly shifted to 368.0 and 374.0 eV, confirming that silver remains mainly in the Ag^+^ chemical state after coupling with CuInS_2_; the small positive binding-energy shift indicates a modified electronic environment around Ag due to interfacial interaction and possible charge redistribution between Ag_3_PO_4_ and CuInS_2_.^35,36^ The high-resolution P 2p XPS spectrum (Fig. 4c) shows a distinct phosphate-related peak at 132.9 eV for pristine Ag_3_PO_4_ and a slightly shifted peak at 133.0 eV for the Ag_3_PO_4_/CuInS_2_ composite, confirming that phosphorus is retained in the P^5+^ state within the PO_4_^3−^ framework of Ag_3_PO_4_ after composite formation.^37^ The high-resolution O 1s XPS spectra (Fig. 4d) show that pristine Ag_3_PO_4_ contains deconvoluted oxygen species at 530.4, 532.0 and 533.0 eV, which can be assigned respectively to lattice oxygen in the Ag–O/P–O framework of Ag_3_PO_4_, surface hydroxyl or chemisorbed oxygen species, and adsorbed H_2_O/weakly bound surface oxygen,^38,39^ while in the Ag_3_PO_4_/CuInS_2_ composite these components shift to about 530.8 and 533.0 eV, indicating that the phosphate oxygen environment is retained after coupling with CuInS_2_. The high-resolution Cu 2p XPS spectrum (Fig. 4e) shows a characteristic Cu 2p_3/2_ peak at 932.2 eV for pristine CuInS_2_, which confirms that copper is mainly present as Cu^+^ within the chalcopyrite CuInS_2_ lattice, while the corresponding peak in the Ag_3_PO_4_/CuInS_2_ composite shifts slightly to 932.0 eV with a broader and lower-intensity profile, indicating that Cu retains its monovalent chemical state after coupling.

**Fig. 4:**
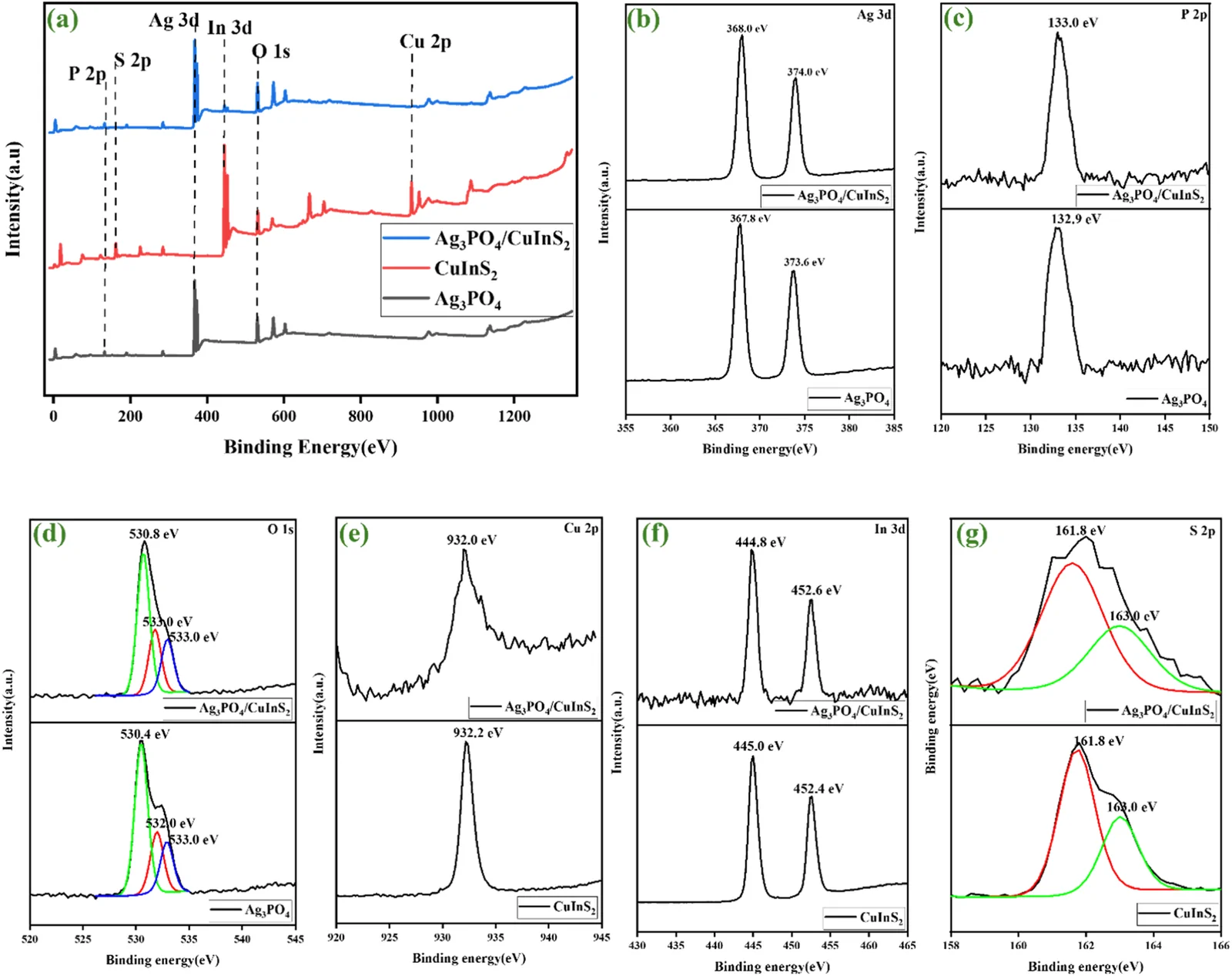
(a) XPS survey spectra of Ag_3_PO_4_, CuInS_2_ and Ag_3_PO_4_/CuInS_2_ with corresponding high-resolution spectra of (b) Ag 3d, (c) P 2p, (d) O 1s, (e) Cu 2p, (f) In 3d and (g) S 2p.

The high-resolution In 3d XPS spectrum (Fig. 4f) shows two characteristic spin–orbit peaks for pristine CuInS_2_ at 445.0 eV and 452.4 eV, assigned to In 3d_5/2_ and In 3d_3/2_, respectively, confirming that indium exists mainly as In^3+^ in the CuInS_2_ lattice, while the Ag_3_PO_4_/CuInS_2_ composite exhibits the corresponding In 3d doublet at 444.8 eV and 452.6 eV, indicating that the indium chemical state is retained after heterojunction formation. The high-resolution S 2p XPS spectra of pristine CuInS_2_ and the Ag_3_PO_4_/CuInS_2_ composite (Fig. 4g) show two deconvoluted peaks at approximately 161.8 and 163.0 eV, assigned to S 2p_3/2_ and S 2p_1/2_ of lattice S^2−^ in CuInS_2_, confirming the successful formation and preservation of the sulfide phase.^39^ The formation of heterostructure as confirmed from the XPS analysis shows that the Ag_3_PO_4_ can provide strong oxidative holes, while CuInS_2_ can improve visible-light harvesting and facilitate charge migration across the interface to suppress rapid electron–hole recombination, promote efficient charge separation, increase the availability of reactive holes, hydroxyl radicals and superoxide species, and ultimately enhance the degradation of organic pollutants under visible-light irradiation.^40^

The XPS peak-fitting parameters, including binding energies (eV), full width at half maximum (FWHM, eV), and integrated peak areas for pristine Ag_3_PO_4_, CuInS_2_, and Ag_3_PO_4_/CuInS_2_ heterostructure, estimated from survey spectra using Gaussian peak fitting are shown in Table S1.

### 3.4 Textural property of the materials

The textural properties of Ag_3_PO_4_, CuInS_2_ and Ag_3_PO_4_/CuInS_2_ heterostructure composite were investigated using nitrogen adsorption–desorption analysis to evaluate their surface area and porosity characteristics as shown in Fig. 5a–d. The isotherms exhibited type IV behaviour with H3-type hysteresis loops, indicating the presence of mesoporous structures, which was further supported by the Barrett–Joyner–Halenda (BJH) pore size distribution showing pore diameters mainly within the 2–50 nm mesoporous range.^41,42^

**Fig. 5:**
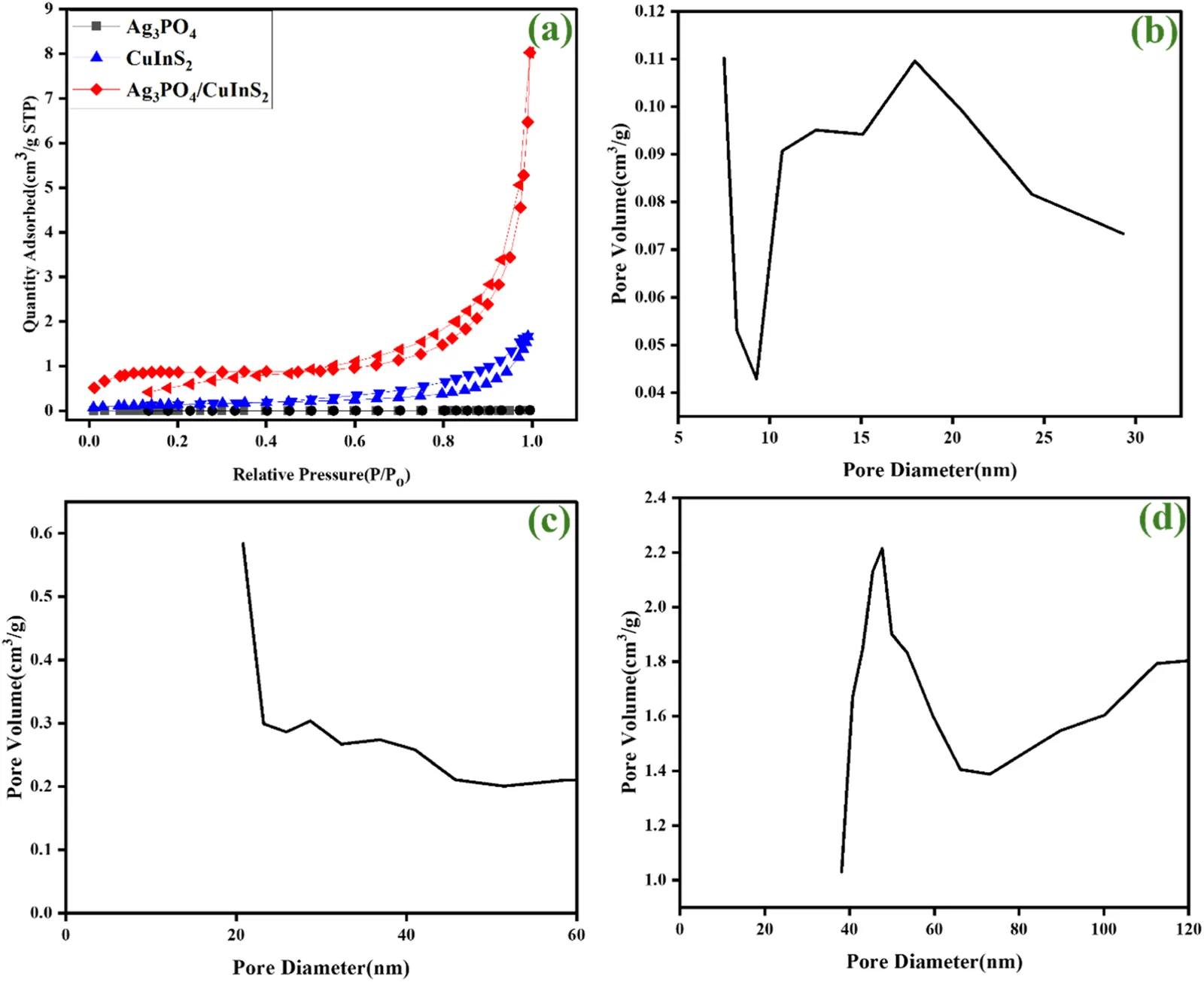
The N_2_ adsorption isotherms for (a) Ag_3_PO_4_, CuInS_2_ and Ag_3_PO_4_/CuInS_2_, and average pores volume and pore size distribution for (b) Ag_3_PO_4_, (c) CuInS_2_ and (d) Ag_3_PO_4_/CuInS_2_ heterostructure composites.

The pristine Ag_3_PO_4_ sample displayed a specific surface area of 6.45 m^2^ g^−1^, an average pore size of 17.90 nm and a total pore volume of 0.10 cm^3^ g^−1^, while CuInS_2_ showed improved textural properties with a surface area of 24.78 m^2^ g^−1^, an average pore size of 32.80 nm and a pore volume of 0.34 cm^3^ g^−1^. Notably, the Ag_3_PO_4_/CuInS_2_ composite exhibited the highest specific surface area, average pore size and total pore volume of 39.22 m^2^ g^−1^, 47.70 nm and 2.21 cm^3^ g^−1^, respectively, confirming that the coupling of Ag_3_PO_4_ with CuInS_2_ significantly enhanced the porosity and surface accessibility of the material (Table 1). This improved mesoporous architecture is beneficial for photocatalytic applications because it provides more accessible active sites, promotes pollutant adsorption, facilitates reactant and intermediate diffusion, and improves interfacial contact between Ag_3_PO_4_ and CuInS_2_ thereby supporting more efficient charge separation and enhanced pollutant degradation under visible-light irradiation^43,44^

**Table 1:** BET surface area pore size and pore volume of photocatalyst material

Photocatalyst materials	*S*_BET_ (m^2^ g^−1^)	Pore diameter (nm)	Pore volume (cm^3^ g^−1^)	Crystalline size (nm)
Ag_3_PO_4_	6.45	17.90	0.10	52.72
CuInS_2_	24.78	32.80	0.34	16.38
Ag_3_PO_4_/CuInS_2_	39.22	47.70	2.21	38.07

### 3.5 Optical analysis of the materials

The optical properties of a photocatalyst play a central role in determining its visible-light activity, since light absorption, bandgap energy and photon utilization directly influence the generation of photogenerated electron–hole pairs required for pollutant degradation.^45^ As shown in Fig. 6, the UV-vis diffuse reflectance spectra show that Ag_3_PO_4_, CuInS_2_ and the Ag_3_PO_4_/CuInS_2_ heterostructure possess distinct optical absorption behaviours, with pristine Ag_3_PO_4_ displaying a sharp absorption edge in the visible region, CuInS_2_ exhibiting broad and intense visible-light absorption due to its narrow bandgap nature, and the Ag_3_PO_4_/CuInS_2_ composite showing an extended absorption response compared with Ag_3_PO_4_, confirming that coupling with CuInS_2_ improves the light-harvesting ability of the Ag_3_PO_4_-based system. The absorption extension observed for the composite indicates successful electronic interaction between Ag_3_PO_4_ and CuInS_2_, which can promote stronger utilization of visible photons during photocatalysis. The corresponding Tauc plot further supports this behaviour, as the optical bandgap of the composite (1.92 eV) appears lower than that of pristine Ag_3_PO_4_ (2.28 eV), suggesting that the incorporation of CuInS_2_ (1.16 eV) narrows the effective optical transition energy and enhances visible-light responsiveness. The lower apparent band gap of the heterostructure relative to Ag_3_PO_4_ suggests improved utilization of longer-wavelength visible light, consistent with the extended absorption observed in the diffuse reflectance spectra. However, the comparatively narrower band gap of CuInS_2_ does not independently guarantee superior photocatalytic activity, since efficient pollutant degradation also requires effective separation and transport of photogenerated charge carriers and suitable oxidation–reduction potentials. This improved optical property is important because efficient photocatalysis depends strongly on the ability of the catalyst to absorb light, generate electron–hole pairs, and transfer these charges before recombination occurs.^46^ Therefore, the broadened visible-light absorption and reduced effective bandgap of Ag_3_PO_4_/CuInS_2_ are expected to increase photogenerated charge production under visible irradiation, while the heterojunction interface can facilitate charge separation between Ag_3_PO_4_ and CuInS_2_, suppress recombination, and improve the formation of reactive species required for pollutant degradation.^47^

**Fig. 6:**
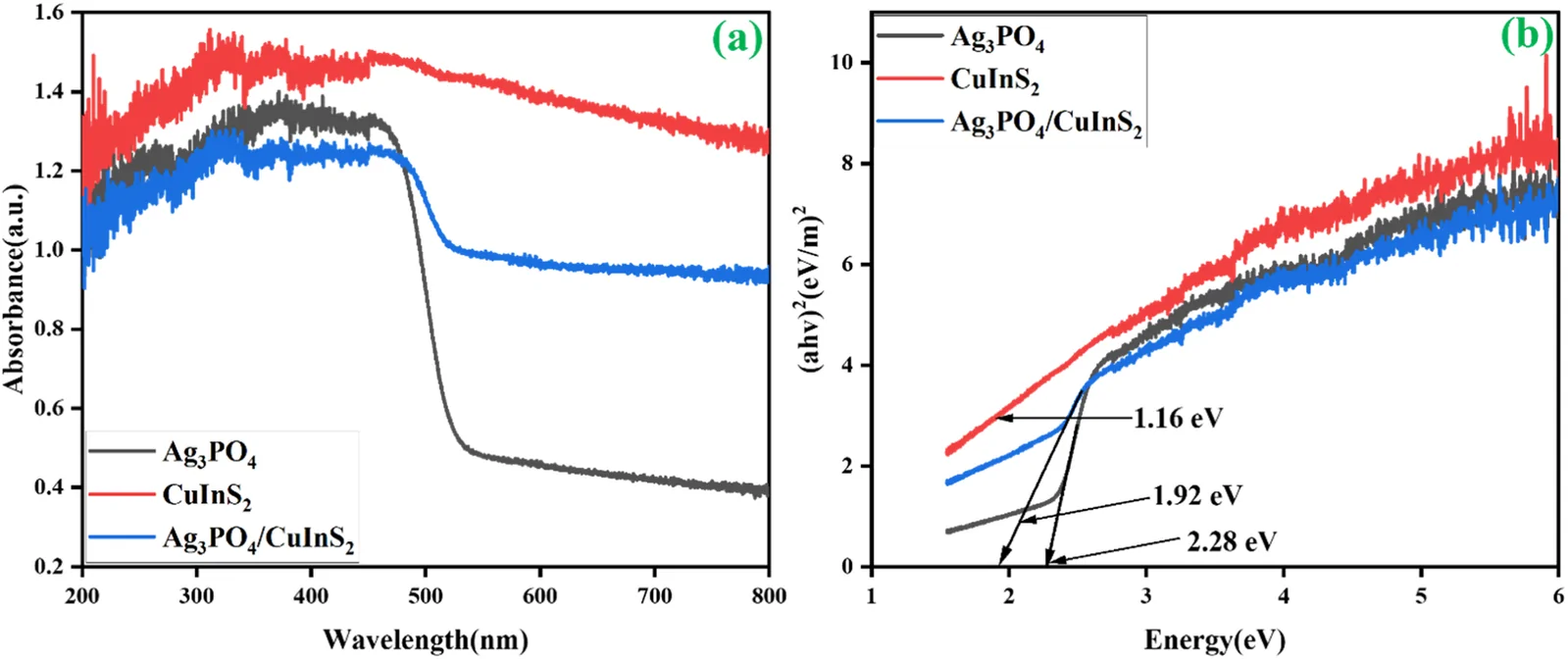
(a) UV-vis DRS spectra along with (b) corresponding Tauc plots of Ag_3_PO_4_, CuInS_2_ and the Ag_3_PO_4_/CuInS_2_ heterostructure.

Analyses of electrochemical impedance spectroscopy and photoluminescence are essential in evaluating the interfacial charge-transfer resistance and electron–hole recombination behaviour, respectively, thereby providing critical insight into the charge-separation efficiency and photocatalytic performance of semiconductor materials.^48^ The electrochemical impedance spectroscopy and photoluminescence results as shown in Fig. 7 collectively demonstrate the improved charge-carrier dynamics of the Ag_3_PO_4_/CuInS_2_ heterostructure. As shown in Fig. 7a, pristine Ag_3_PO_4_ exhibits the largest Nyquist semicircle, indicating high interfacial charge-transfer resistance and relatively slow migration of photogenerated carriers, while CuInS_2_ shows a smaller arc radius. The Ag_3_PO_4_/CuInS_2_ composite displays the smallest semicircle, confirming that heterojunction formation reduces charge-transfer resistance and promotes more efficient electron transport across the Ag_3_PO_4_–CuInS_2_ interface. Consistent with this observation, the PL spectra in Fig. 7b exhibit a prominent emission peak centred at around 350 nm for all three materials. Pristine Ag_3_PO_4_ shows the highest PL intensity, reflecting rapid radiative recombination of photogenerated electron–hole pairs, whereas CuInS_2_ exhibits a lower emission intensity. Notably, the Ag_3_PO_4_/CuInS_2_ heterostructure shows the strongest PL quenching, indicating that interfacial coupling effectively suppresses charge-carrier recombination and prolongs the lifetime of photogenerated electrons and holes. The reduced impedance and weakened PL emission of the composite therefore confirm enhanced interfacial charge separation and migration, which increase the availability of photogenerated charge carriers for reactive-species formation and contribute to its superior visible-light-driven photocatalytic degradation of ciprofloxacin.^49,50^

**Fig. 7:**
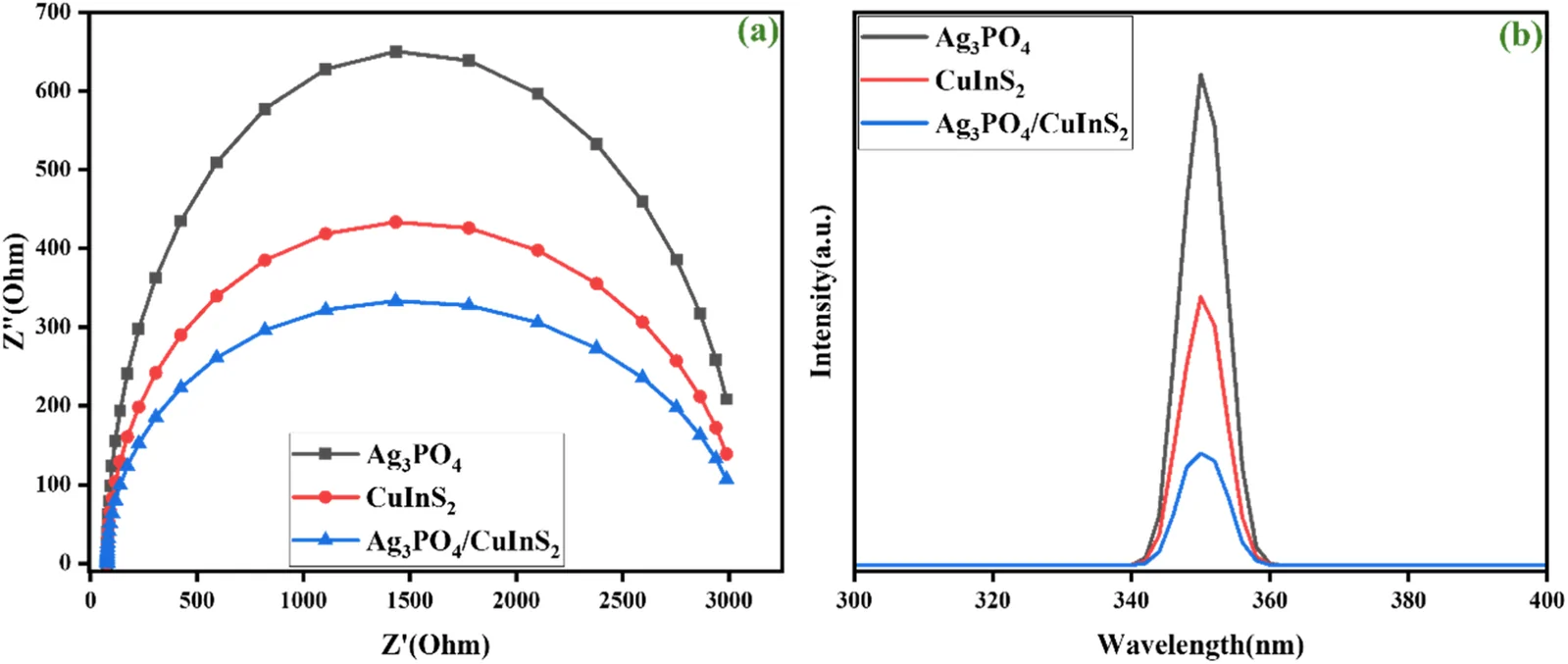
(a) EIS analysis and (b) PL spectra of the synthesized Ag_3_PO_4_, CuInS_2_ and the Ag_3_PO_4_/CuInS_2_ heterostructure composites.

In summary to the characterization results as stated above, the hierarchical flower-like architecture of the Ag_3_PO_4_/CuInS_2_ heterostructure provides several structural advantages that contribute to its enhanced photocatalytic performance. Unlike the relatively smooth and compact faceted particles of pristine Ag_3_PO_4_, the composite consists of interconnected CuInS_2_ flakes closely associated with Ag_3_PO_4_ particles, producing a rough and porous structure with increased surface accessibility and intimate semiconductor interfaces. The importance of this arrangement is supported by the BET results (Fig. 5 and Table 1), which reveal a significantly higher specific surface area of 39.22 m^2^ g^−1^ for the heterostructure compared with 6.45 and 24.78 m^2^ g^−1^ for pristine Ag_3_PO_4_ and CuInS_2_, respectively. The enlarged surface area and accessible pore structure can increase the availability of photocatalytically active sites, favour ciprofloxacin adsorption and facilitate molecular diffusion toward reactive surfaces. Furthermore, the interconnected hierarchical structure may promote internal light scattering and redistribution of incident photons, potentially increasing photon utilization, while the incorporation of CuInS_2_ extends visible-light absorption, as supported by the UV-vis DRS results (Fig. 6). More importantly, the intimate contact between the flower-like CuInS_2_ assemblies and faceted Ag_3_PO_4_ particles provides interfacial pathways for photogenerated charge migration, thereby reducing charge-transfer resistance and limiting electron–hole recombination. This interpretation is consistent with the reduced EIS semicircle and suppressed PL emission observed for the heterostructure (Fig. 7). Consequently, the improved photocatalytic activity can be attributed to the combined influence of enhanced surface accessibility, broadened visible-light absorption, and favourable interfacial charge-transfer characteristics rather than morphology alone.

### 3.6 Photocatalytic activity measurement

The photocatalytic properties of Ag_3_PO_4_, CuInS_2_ and the Ag_3_PO_4_/CuInS_2_ heterostructure composites were assessed *via* CIP photodegradation tests with visible light. As displayed in Fig. 8a, CIP can hardly be removed in the absence of photocatalyst materials with visible light irradiation while following a 30 min period of dark reaction, all the synthesized materials exhibited certain adsorption capacities toward CIP molecules. Upon light illumination for 60 min, the CIP removal efficiencies reached 44.30%, 58.87%, and 96.13% for Ag_3_PO_4_, CuInS_2_ and the Ag_3_PO_4_/CuInS_2_ heterostructure composites. The enhanced activity of the Ag_3_PO_4_/CuInS_2_ heterostructure composite can be attributed to the successful formation of a heterojunction between Ag_3_PO_4_ and CuInS_2_, which likely improved visible-light absorption, promoted interfacial charge transfer, and suppressed rapid recombination of photogenerated electron–hole pairs. The comparative analysis presented in Table S2 highlights differences in photocatalytic efficiency, irradiation time, catalyst dosage, and experimental conditions among previously reported semiconductor-based photocatalysts.^51–57^ The Ag_3_PO_4_/CuInS_2_ heterostructure demonstrated competitive performance, achieving 96.13% CIP degradation within 60 min under visible-light irradiation at a relatively low catalyst loading of 0.05 g L^−1^.

**Fig. 8:**
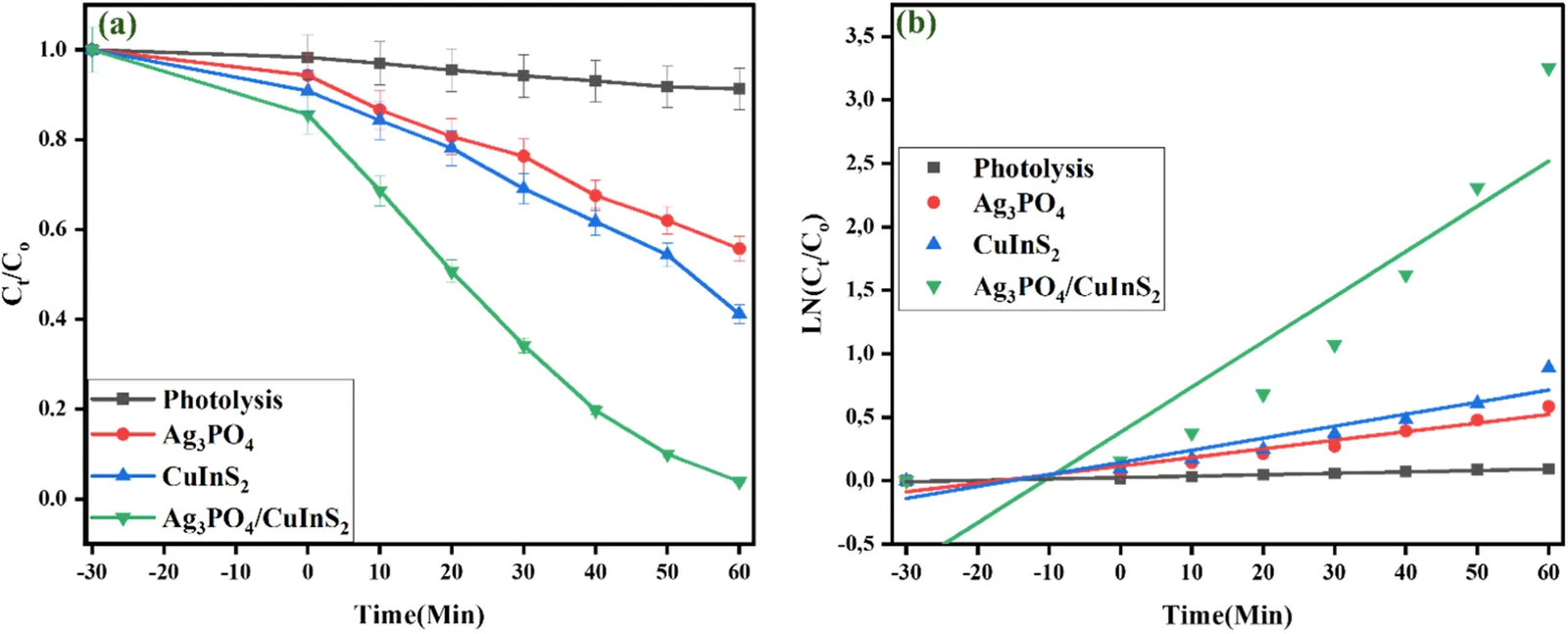
(a) Photocatalytic CIP degradation and (b) kinetic curves applying Ag_3_PO_4_, CuInS_2_ and the Ag_3_PO_4_/CuInS_2_ materials (experiment conditions: light intensity: 142.86 W m^−2^; initial CIP concentration: 7 mg L^−1^; catalyst dosage: 50 mg; initial pH: 6.6).

In Fig. 8b, the corresponding first-order kinetic rate constant (*k*) for the Ag_3_PO_4_/CuInS_2_ heterostructure composite is 0.03561 min^−1^, 5.3 and 3.76 times that of Ag_3_PO_4_ (*k* = 0.00678 min^−1^) and CuInS_2_ (*k* = 0.00948 min^−1^), respectively as compiled in Table S3. This improvement in the kinetic rate of the heterostructure Ag_3_PO_4_/CuInS_2_ can be attributed to the coupling of Ag_3_PO_4_ with CuInS_2_ which significantly accelerated the degradation reaction through synergistic interaction between the two semiconductors which created more efficient reactive species generation for CIP oxidation.

As shown in Fig. 9a, increasing the Ag_3_PO_4_/CuInS_2_ dosage from 10 to 50 mg improved CIP degradation from 76.80% to 96.12% after 60 min of visible-light irradiation. This improvement can be attributed to the greater availability of photocatalytically active sites, allowing more CIP molecules to interact with photogenerated charge carriers and reactive oxygen species. However, further increases in catalyst dosage to 70 and 100 mg reduced degradation efficiency to 90.31% and 73.02%, respectively. At higher catalyst concentrations, increased light scattering and attenuation can limit photon penetration through the suspension, resulting in insufficient irradiation of particles located farther from the light-exposed region. In addition, increased particle–particle interactions may favour aggregation, reducing the accessibility of some reactive surface sites.^58,59^ Consequently, the increase in catalyst quantity no longer produces a corresponding increase in effectively illuminated reaction sites. The maximum degradation efficiency at 50 mg therefore reflects a favourable balance between the availability of catalytic surface sites and photon penetration under the investigated conditions. These limitations could potentially be addressed by employing photoreactors with shorter optical path lengths, improved mixing, and more uniform light distribution, allowing effective irradiation of catalytic surfaces without excessive catalyst consumption while the immobilization of Ag_3_PO_4_/CuInS_2_ onto suitable supports could improve catalyst handling, reduce particle aggregation, and facilitate recovery.^60,61^

**Fig. 9:**
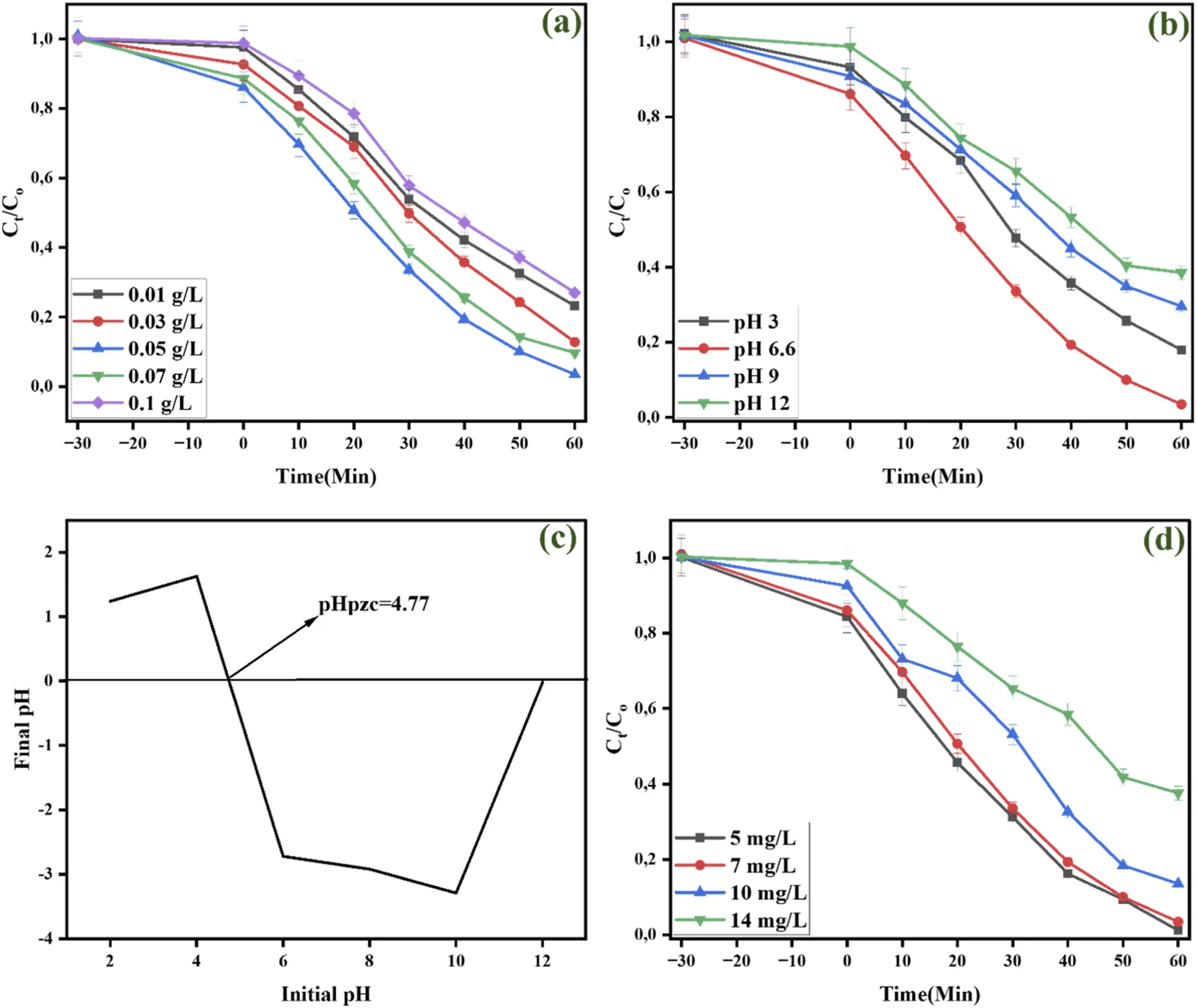
(a) Sample dosage, (b) effect of pH, (c) point of zero charge and (d) initial CIP concentration on the removal performance of CIP using Ag_3_PO_4_/CuInS_2_ heterostructure composites.

The effect of pH on ciprofloxacin degradation is governed by the relationship between CIP speciation and the surface charge of the photocatalyst.^62^ CIP has two principal p*K*_a_ values of approximately 6.1 and 8.7, existing mainly as a cation below pH 6.1, a zwitterion between pH 6.1 and 8.7, and an anion above pH 8.7.^63^ Since the catalyst has a point of zero charge of 4.77 as shown in Fig. 9c, its surface is positively charged below this pH and negatively charged above it. Accordingly, the lower degradation observed at pH 3 is attributed to electrostatic repulsion between positively charged CIP and the positively charged catalyst surface. At pH 6.6, CIP is predominantly zwitterionic, while the catalyst surface is negatively charged, allowing favourable interaction between the protonated piperazinyl group of CIP and the catalyst surface, which promotes adsorption and results in the highest degradation efficiency.^64^ At pH 9 and 12 (Fig. 9b), both CIP and the catalyst surface are negatively charged, leading to increasing electrostatic repulsion, reduced surface interaction and lower degradation. Although the Ag_3_PO_4_/CuInS_2_ heterostructure exhibited maximum CIP degradation efficiency at pH 6.6, its sustained photocatalytic activity across the investigated pH range (3–12) suggests a degree of tolerance to variations in solution pH. This behaviour indicates its potential applicability under realistic wastewater conditions, where pH fluctuations may arise from changes in wastewater composition and treatment processes.^65^

Photocatalytic degradation involves the absorption of pollutant molecules onto the catalyst surface, their reaction with photogenerated charge carriers and reactive species, and the subsequent desorption of degradation products.^66^ As shown in Fig. 9d, the degradation efficiency of CIP over the Ag_3_PO_4_/CuInS_2_ heterostructure decreased progressively as the initial CIP concentration increased from 5 to 14 mg L^−1^. Approximately 98.81% degradation was achieved at 5 mg L^−1^, whereas the efficiency declined to about 62.51% at 14 mg L^−1^ after 60 min of irradiation. At lower concentrations, the available active sites and reactive species were sufficient to interact effectively with CIP molecules, resulting in rapid degradation. In contrast, increasing the CIP concentration caused greater occupation of the catalyst surface and increased competition for a limited number of active sites and photogenerated reactive species. Moreover, the accumulation of degradation intermediates may have further blocked surface-active sites and competed with the parent CIP molecules for adsorption. Higher pollutant concentrations may also reduce light penetration through the solution, thereby limiting photon absorption by the photocatalyst. These effects collectively account for the decrease in CIP degradation efficiency observed at elevated initial concentrations.

The reproducibility and durability of Ag_3_PO_4_/CuInS_2_ composite in the potential practical application was demonstrated over four consecutive photocatalytic cycles under identical reaction conditions. As shown in Fig. 10a, the degradation efficiency decreased moderately from 96.13% in the first cycle to 89.45% after the fourth cycle, corresponding to a retention of approximately 93.1% of the initial activity. This limited reduction indicates that the composite possesses good photocatalytic stability and can be reused without substantial loss of performance. The slight decline may be attributed to catalyst loss during recovery and washing, partial blockage of active sites by adsorbed intermediates, and particle agglomeration during repeated irradiation.^67^ Nevertheless, the high degradation efficiency maintained after four cycles demonstrates the promising operational durability of the Ag_3_PO_4_/CuInS_2_ heterostructure for repeated CIP treatment. The post-degradation SEM image in Fig. 10b shows that the catalyst largely retained its hierarchical cauliflower-like morphology, with only modest agglomeration and surface roughening, indicating reasonable morphological stability after photocatalytic use. The diffraction patterns exhibit nearly identical characteristic peak positions, indicating that the crystalline structure was largely preserved after four consecutive degradation cycles. Furthermore, the absence of clearly detectable new diffraction peaks attributable to metallic Ag suggests that substantial photocorrosion and Ag^+^ reduction was not evident within the detection limits of XRD Fig. S2. These findings, together with the retention of approximately 93.1% of the initial photocatalytic activity, demonstrate promising short-term structural stability and reusability of the Ag_3_PO_4_/CuInS_2_ heterostructure. For long-term applications, catalyst immobilization on suitable supports, improved recovery methods, optimization of operating conditions, and appropriate interfacial modifications may minimize material losses, reduce agglomeration, and potentially suppress photocorrosion of the Ag_3_PO_4_/CuInS_2_ heterostructure under continuous wastewater treatment conditions.

**Fig. 10:**
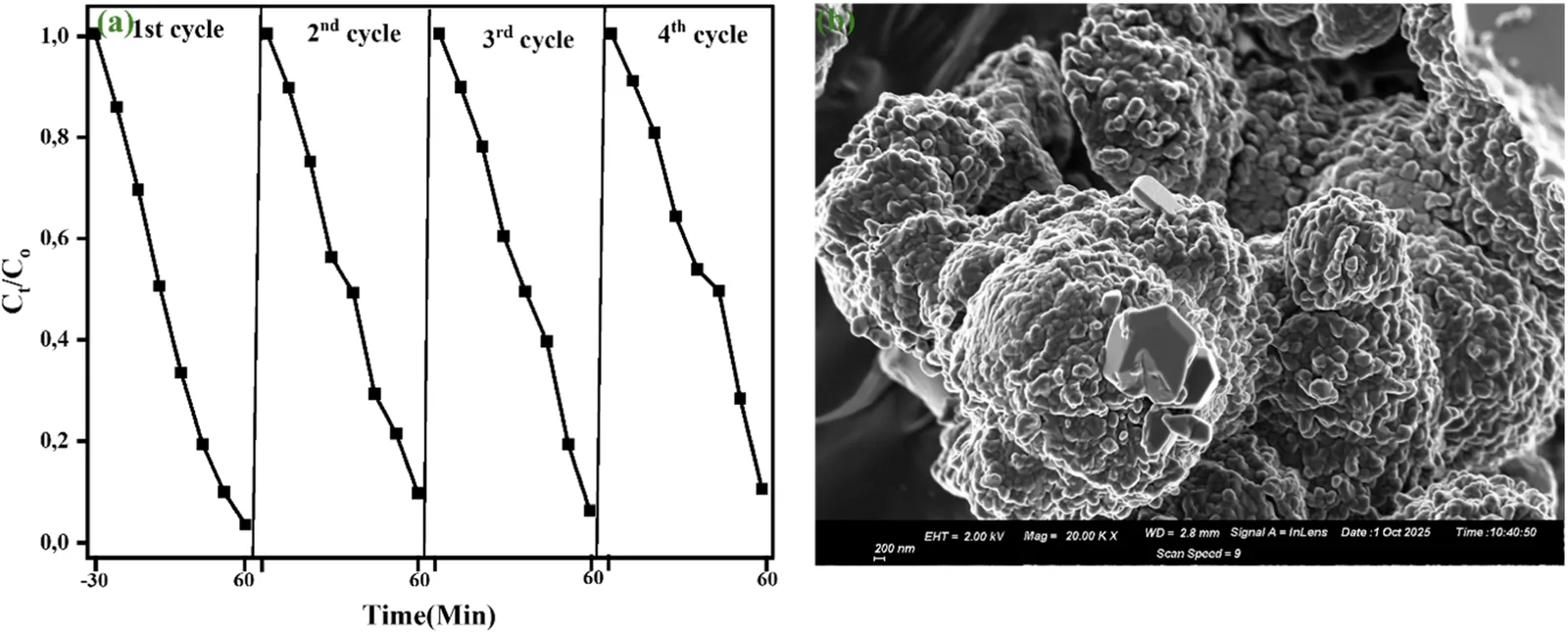
(a) Recyclability performance of the photocatalyst over successive degradation cycles and (b) post-degradation SEM image showing the retention of its hierarchical cauliflower-like morphology after reuse.

### 3.7 Proposed photocatalytic degradation pathway of ciprofloxacin

The Liquid Chromatography-Mass Spectrometry (LC-MS) was used to evaluate the photocatalytic transformation pathway of ciprofloxacin (CIP) under visible light irradiation using the synthesized Ag_3_PO_4_/CuInS_2_ heterostructure composite (Fig. S3). Under visible-light irradiation, the photocatalyst absorbs photons with energy equal to or greater than its band gap, resulting in the excitation of electrons from the valence band to the conduction band and the simultaneous formation of photogenerated holes. The conduction-band electrons may react with dissolved molecular oxygen to generate superoxide radicals (˙O_2_^−^), while the photogenerated holes can directly oxidize CIP or promote the formation of hydroxyl radicals (˙OH) through reactions involving surface-adsorbed water or hydroxide ions. These reactive species subsequently attack the electron-rich functional groups of CIP, particularly the nitrogen atoms and α-carbon positions of the piperazine ring, as well as susceptible sites within the quinolone framework.^68,69^ Ciprofloxacin is normally detected in positive-ionization mode as the protonated molecular ion at approximately *m*/*z* 332.14. The absence of this parent ion in the spectrum obtained after 60 min indicates substantial transformation of the original CIP molecule. The initial degradation stage is proposed to involve hydroxylation and oxidation of the piperazine moiety, which may generate an oxygenated intermediate represented tentatively by an ion around *m*/*z* 362 (Fig. 11). Oxidative attack on the tertiary nitrogen and adjacent carbon atoms can then promote *N*-dealkylation, producing intermediates such as those represented by *m*/*z* 344. Continued oxidation weakens the C–N bonds within the piperazine ring, leading to ring opening and the formation of a partially oxidized side-chain intermediate tentatively assigned to *m*/*z* 334. These initial transformations are expected to occur rapidly and may therefore produce transient intermediates that are not strongly represented in the spectrum recorded after 60 min.^69^

**Fig. 11:**
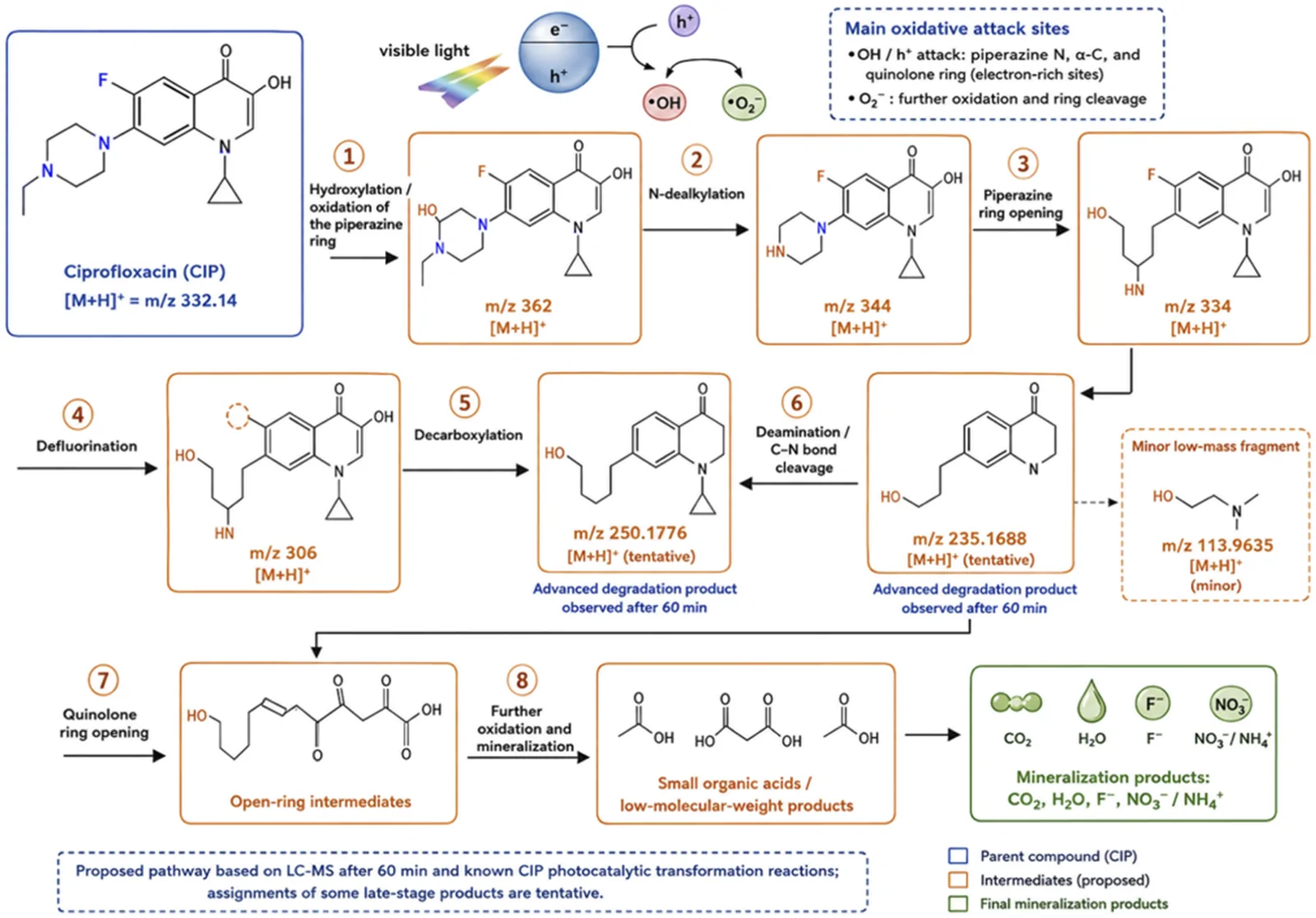
Tentative transformation pathways proposed for ciprofloxacin during visible-light photocatalytic treatment with the Ag_3_PO_4_/CuInS_2_ heterostructure based on the detected LC-MS signals and plausible reaction steps.

Further oxidation of the opened piperazine chain can result in successive deamination, cleavage of C–N bonds and removal of small nitrogen-containing fragments. Defluorination may also occur through oxidative substitution or cleavage of the aromatic C–F bond, producing a lower-molecular-weight intermediate tentatively represented by *m*/*z* 306. At this stage, hydroxyl radicals and photogenerated holes may attack both the remaining side chain and the quinolone nucleus. The removal of the carboxyl group through decarboxylation, together with additional deamination and side-chain cleavage, is proposed to yield advanced transformation products represented by the experimentally observed ions at *m*/*z* 250.1776 and 235.1688. The high relative intensity of the *m*/*z* 235.1688 ion suggests that this product accumulated during the 60 min treatment period and may therefore represent a comparatively stable late-stage intermediate transformation product.^70^ The minor ion observed at *m*/*z* 113.9635 may be associated with the formation of a low-molecular-weight fragment following cleavage of the oxidized piperazine-derived side chain. Similarly, the signals at *m*/*z* 371.3280, 409.1360 and those above *m*/*z* 500 were not incorporated into the main proposed pathway because they may arise from adduct formation, molecular clusters, background contaminants components.^70^ Following the formation of the *m*/*z* 250.1776 and 235.1688 intermediates, continued attack by ˙OH, h^+^ and, to a lesser extent, ˙O_2_^−^ is proposed to initiate cleavage of the quinolone ring. Ring-opening reactions can generate unstable aliphatic compounds containing hydroxyl, carbonyl and carboxyl functional groups. These species are subsequently oxidized into smaller organic acids and other low-molecular-weight products. Prolonged photocatalytic treatment may ultimately lead to partial mineralization, producing CO_2_ and H_2_O, together with inorganic fluoride and nitrogen-containing ions such as F^−^, NO_3_^−^ and NH_4_^+^. Overall, the proposed pathway indicates that CIP degradation proceeds mainly through oxidative modification of the piperazine group, followed by *N*-dealkylation, piperazine-ring opening, defluorination, decarboxylation, deamination and destruction of the quinolone framework. The ions at *m*/*z* 250.1776 and 235.1688 provide evidence for the formation of advanced degradation products after 60 min.

Following the identification of CIP transformation products by LC-MS, TOC analysis was performed to determine the extent of their subsequent mineralization. As shown in Fig. S4, the Ag_3_PO_4_/CuInS_2_ composite achieved approximately 59.5% TOC removal, substantially outperforming Ag_3_PO_4_ (17.65%) and CuInS_2_ (23.09%), thereby confirming enhanced CIP mineralization; however, the lower TOC removal relative to the 96.13% CIP degradation indicates that some organic intermediates remained after irradiation. The remaining organic intermediates could be further mineralized by extending the irradiation time thereby allowing sustained attack by the generated reactive species and promoting their conversion into CO_2_, H_2_O, and inorganic ions.

### 3.8 Ecotoxicity of ciprofloxacin

The ecotoxicological data (Table S4) indicates that CIP exerts pronounced toxicity on primary producers, particularly freshwater algae and aquatic macrophytes, thereby identifying these organisms as the most sensitive trophic level within aquatic ecosystems. Among the tested species, *Microcystis aeruginosa* was the most sensitive, with a NOEC of 0.0079 mg L^−1^ after 120 h of exposure, demonstrating marked inhibition of growth and photosynthetic activity at environmentally relevant concentrations.^71^ Likewise, *Microcystis panniformis* (NOEC = 0.01358 mg L^−1^) and *Anabaena flos-aquae* (NOEC = 0.0102 mg L^−1^) exhibited pronounced growth inhibition, corroborating the high sensitivity of cyanobacteria to fluoroquinolone antibiotics.^72,73^ Green algae, such as *Pseudokirchneriella subcapitata* (NOEC = 1.10–2.0 mg L^−1^) and *Chlorella vulgaris* (EC_50_ = 9.23 mg L^−1^), also showed reductions in growth, chlorophyll synthesis and biomass accumulation, albeit at comparatively higher concentration ranges.^71,74,75^ Aquatic macrophytes, including *Lemna minor* (NOEC = 0.053–0.24 mg L^−1^) and *Spirodela polyrhiza* (NOEC = 0.28 mg L^−1^), displayed significant decreases in frond number, biomass and growth rate under chronic exposure, suggesting that CIP may impair ecosystem primary productivity and nutrient cycling through deleterious effects on photosynthetic primary producers.^76^

In contrast, aquatic invertebrates and fish generally exhibited lower acute sensitivity to CIP, although pronounced chronic and sublethal effects were documented. Acute toxicity assays with *Daphnia magna* reported minimal mortality at concentrations up to 10 mg L^−1^,^71^ whereas higher exposure levels elicited immobilization and mortality, with EC_50_ values ranging from 14–73 mg L^−1^.^77^ Prolonged exposure substantially increased toxicity, with chronic NOEC values as low as 0.056 mg L^−1^ associated with reduced reproductive output, impaired somatic growth, delayed maturation and diminished offspring production.^78^ Moreover, environmentally relevant concentrations (0.0001–0.1 mg L^−1^) induced oxidative stress, glutathione-*S*-transferase activation, cholinesterase inhibition, alterations in antioxidant enzyme activities, behavioural impairments and the production of smaller neonates in *D. magna*, demonstrating that sublethal biochemical and physiological disturbances can occur well below concentrations required to induce mortality.^79^*Ceriodaphnia dubia* appeared more sensitive than *D. magna*, exhibiting increased mortality, reduced survival and impaired reproduction at concentrations between 3 and 4 mg L^−1^.^77^ Fish species, including *Pimephales promelas*, *Gambusia holbrooki* and *Danio rerio*, generally showed low acute toxicity, with limited or no mortality observed at concentrations up to or exceeding 10 mg L^−1^, although adverse effects on embryonic development and larval growth were reported in some instances.^71,74,80^ These data indicate that CIP poses the greatest ecological risk to photosynthetic microorganisms and aquatic macrophytes, while long-term exposure can also adversely affect the health, reproductive capacity and population viability of aquatic invertebrates despite their comparatively lower acute sensitivity.

### 3.9 Risk assessment

The ecological risk assessment based on risk quotient (RQ) values (Fig. 12) reveals pronounced interspecific differences in the sensitivity of aquatic organisms to CIP, with algae consistently representing the most susceptible taxonomic group under both acute and chronic exposure conditions. According to the widely applied RQ classification scheme (RQ < 0.1 = insignificant risk; 0.1 ≤ RQ < 1 = low risk; 1 ≤ RQ < 10 = moderate risk; RQ ≥ 10 = high risk).^81,82^ The majority of monitored sites worldwide presented negligible to low ecological risks for fish and *Daphnia*. In contrast, multiple locations exceeded the thresholds for moderate and high ecological risk for algae. Notably, algal RQ values >10 under both acute and chronic exposure scenarios at the two sites sampled in India, indicating a substantial potential for adverse ecological effects. In comparison, the maximum acute and chronic RQ values for *Daphnia* remained >5, and fish consistently exhibited RQ values below unity at all sampling locations, suggesting a comparatively lower vulnerability of vertebrates. These patterns concur with previous investigations similar to the discussion in Section 3.8 show that primary producers constitute the most sensitive trophic group to fluoroquinolone antibiotics. CIP selectively inhibits bacterial DNA gyrase and topoisomerase IV, enzymes that are evolutionarily related to those present in chloroplasts of photosynthetic microorganisms, thereby disrupting algal growth and photosynthetic efficiency.^83,84^ The elevated ecological risks identified for algae are further explicable by the widespread occurrence and environmental persistence of ciprofloxacin in aquatic systems. Due to its high chemical stability,^6^ incomplete removal in conventional wastewater treatment processes,^85^ and strong sorption to suspended particulate matter and sediments, CIP is continuously discharged into receiving water bodies, resulting in chronic low-level exposure of aquatic biota.^86–88^ Although fish and *Daphnia* generally exhibited insignificant to moderate risks in the present assessment, the limited number of sites where *Daphnia* RQ values exceeded unity indicates that elevated environmental concentrations may adversely affect reproduction, growth, and population dynamics of aquatic invertebrates. Consequently, the substantially lower RQ values for fish compared with algae and *Daphnia* are consistent with previous ecotoxicological studies demonstrating that vertebrates generally display higher toxicity thresholds for fluoroquinolones than lower trophic-level organisms. Hence, algae emerge as the most conservative indicator taxon for environmental risk assessment of antibiotic contamination.^89–91^

**Fig. 12:**
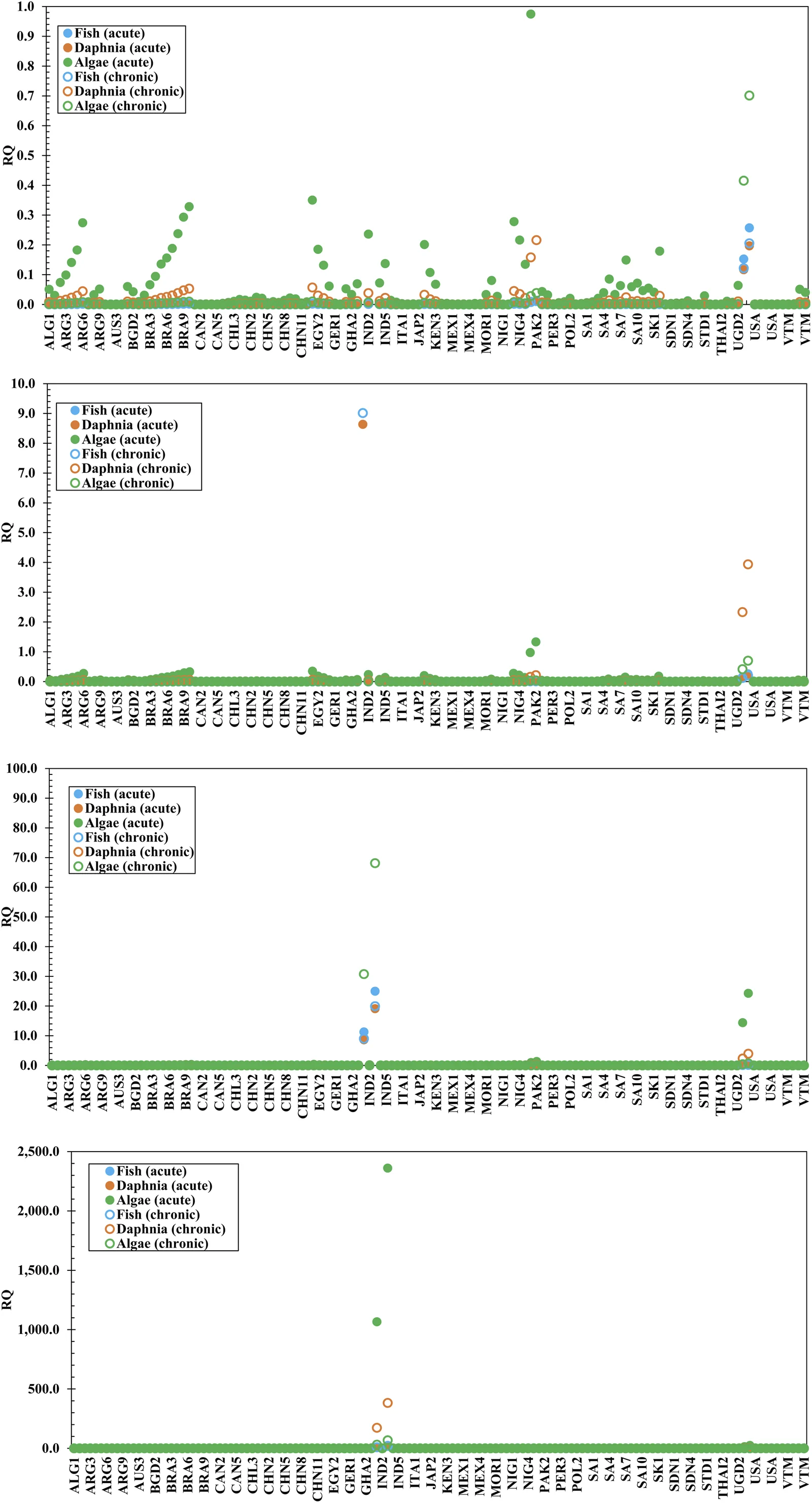
RQs for surface water matrices calculated using acute (full circles) and chronic (open circles) toxicity of ciprofloxacin to fish (blue), algae (green), and *Daphnia* (orange) based on reported MEC of ciprofloxacin globally.

The occurrence of high-risk hotspots for algal communities has substantial ecological implications, as phytoplankton represent the basal trophic level of aquatic food webs and contribute markedly to primary production, nutrient cycling, and oxygen generation.^92^ Sustained inhibition of these communities may therefore precipitate cascading ecological consequences, including decreased biomass production, shifts in community composition, and reduced ecosystem resilience. The predominance of moderate to high algal risk levels identified in this study underscores the urgent necessity for advanced wastewater treatment technologies capable of achieving complete degradation and mineralization of ciprofloxacin prior to effluent discharge. Conventional wastewater treatment processes are typically inadequate for the effective removal of fluoroquinolones, whereas visible-light-driven AOPs such as the Ag_3_PO_4_/CuInS_2_ heterostructured photocatalyst developed herein constitute a promising approach to mitigating the ecological risks posed by persistent antibiotic pollutants. By enhancing degradation efficiency under solar irradiation, this system can reduce the long-term exposure of sensitive aquatic organisms to ciprofloxacin and related contaminants. The ecological risk assessment demonstrates why ciprofloxacin occurrence in aquatic environments requires effective control. Risk quotients calculated from reported environmental concentrations and literature-derived toxicity thresholds indicate that sensitive aquatic organisms, particularly algae and other primary producers, may experience elevated risks in some locations. However, this assessment describes the potential risk associated with environmentally occurring ciprofloxacin and does not demonstrate that photocatalytic treatment eliminated such risk. The toxicity, environmental persistence and residual antibacterial activity of the treated solution and proposed transformation products were not directly determined. Consequently, the high degradation of the parent ciprofloxacin compound should not be equated with an equivalent reduction in ecological risk. Post-treatment toxicity and antimicrobial-activity assays are needed to establish whether the photocatalytic process produces a genuinely safer effluent.

## 4 Proposed degradation mechanism

To investigate the photocatalytic mechanism of Ag_3_PO_4_/CuInS_2_ heterostructure composites, radical quenching experiments were carried out under visible light to identify the reactive species during the photocatalytic reaction, quenching tests were performed using isopropyl alcohol (IPA), 1,4-benzoquinone (BQ) and triethanolamine (TEOA) as scavengers for ˙OH, ˙O_2_^−^ and h^+^, respectively. In the absence of scavengers, photocatalytic degradation efficiency reached 96.13%, while upon the addition of isopropanol, which scavenges hydroxyl radicals, the degradation efficiency decreased to 54.93%, representing a reduction of 41.20 percentage points. The presence of 1,4-benzoquinone resulted in a comparatively smaller decrease to 80.00%, indicating that superoxide radicals contributed to the degradation process but were not the dominant reactive species. The most pronounced inhibition was observed after the addition of triethanolamine, with the degradation efficiency declining to 38.51%, confirming that photogenerated holes and hydroxyl radicals played the predominant role while ˙O_2_^−^ plays a secondary role in CIP oxidation as displayed in Fig. 13.

**Fig. 13:**
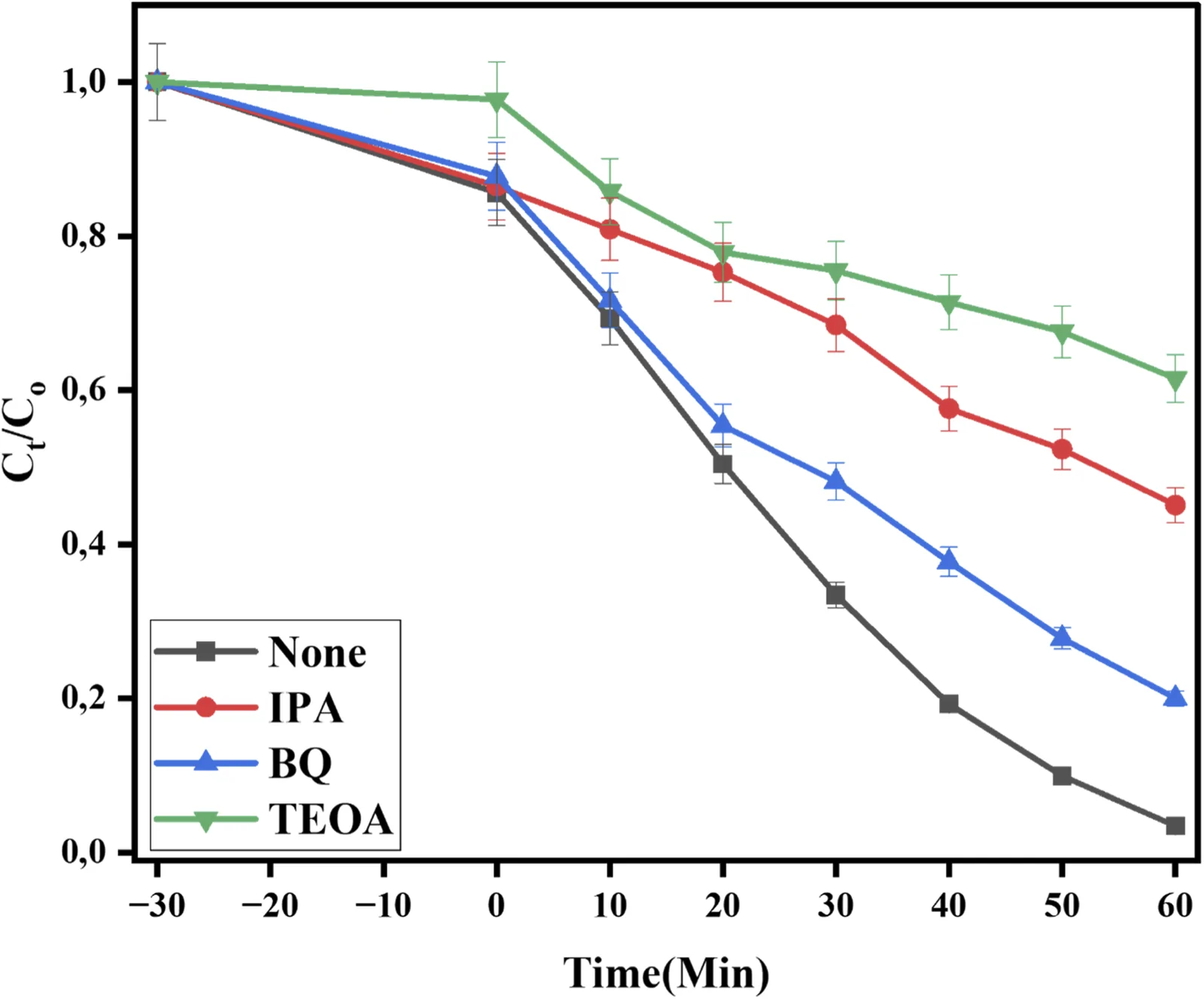
Visible-light photocatalytic degradation of CIP by Ag_3_PO_4_/CuInS_2_ heterostructure in the presence of different scavengers.

In addition to the results of the scavenger experiment, the band-edge positions obtained from the UV-DRS analysis and the EIS and PL result which prove that there is an interfacial interaction between Ag_3_PO_4_ and CuInS_2_, a direct Z-scheme pathway is more plausible for the Ag_3_PO_4_/CuInS_2_ heterostructure than a S-scheme-like charge-transfer or conventional type-II mechanism. Under visible-light irradiation as shown in Fig. 14, both Ag_3_PO_4_ and CuInS_2_ generate electron–hole pairs with the photogenerated electrons in the conduction band of Ag_3_PO_4_ at +0.32 eV preferentially recombine with holes in the valence band of CuInS_2_ at +0.89 eV through interfacial contact. This pathway preserves the electrons with the stronger reduction ability in the CuInS_2_ conduction band at −0.27 eV and the holes with the stronger oxidation ability in the Ag_3_PO_4_ valence band at +2.60 eV. In contrast, a type-II pathway would accumulate electrons in the Ag_3_PO_4_ conduction band and holes in the CuInS_2_ valence band, substantially weakening the redox capacity of the composite while S-scheme would require direct evidence of Fermi-level equilibration, interfacial band bending and the associated internal electric field, which cannot be determined from the present UV-DRS, PL, EIS and trapping measurements alone, hence, Z-scheme configurations are commonly proposed when interfacial charge transfer maintains the strongly oxidizing and reducing carriers of the constituent semiconductors.^93–95^

**Fig. 14:**
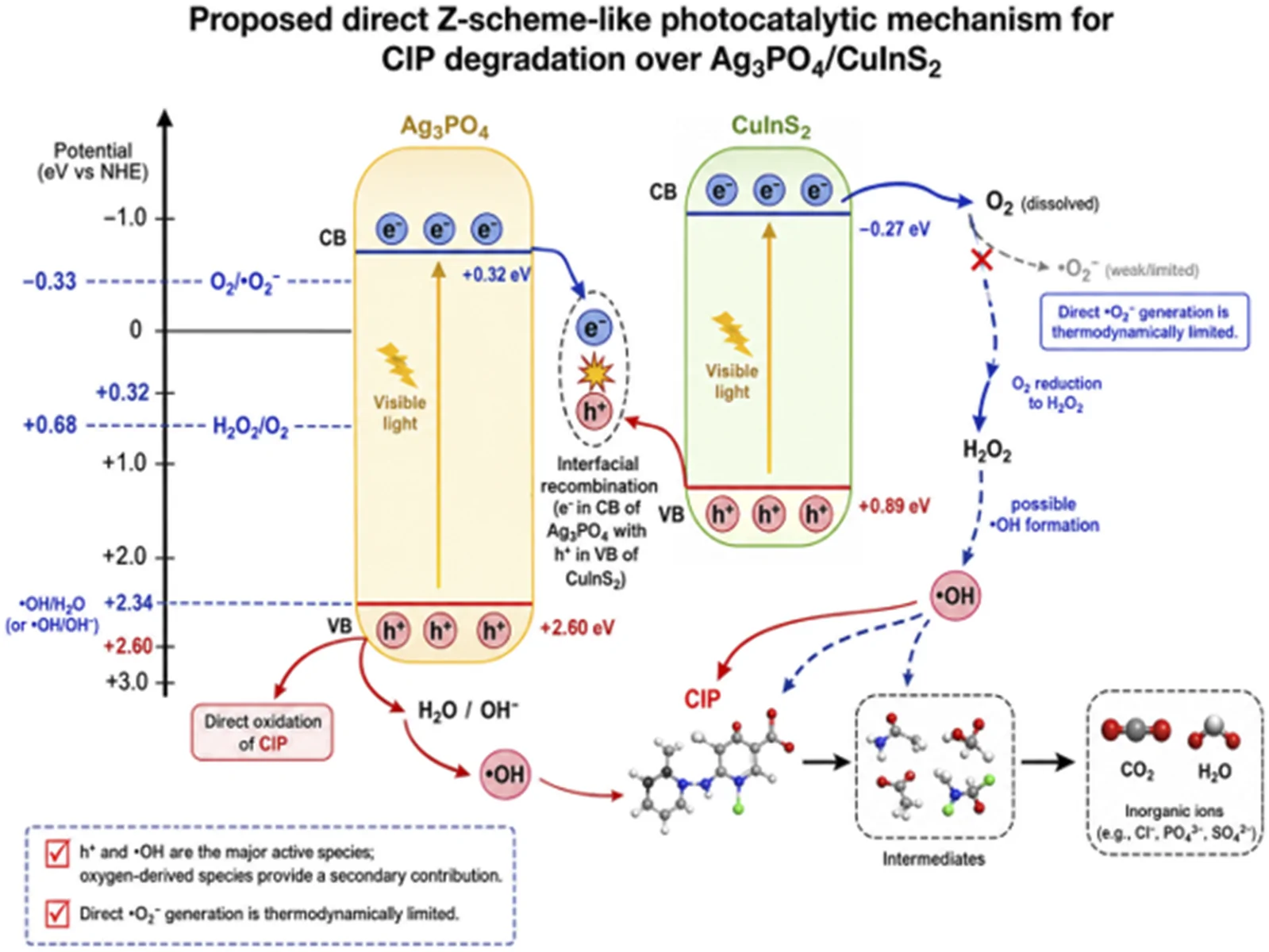
Proposed mechanism for enhanced photocatalytic oxidative degradation of CIP using Ag_3_PO_4_/CuInS_2_ heterostructure composites.

The holes retained in the Ag_3_PO_4_ valence band can oxidize CIP directly and can also convert adsorbed water or hydroxide ions into hydroxyl radicals because the Ag_3_PO_4_ valence-band potential of +2.60 eV is more positive than the oxidation potential required for hydroxyl-radical formation. The electrons remaining in the CuInS_2_ conduction band at −0.27 eV are slightly less negative than the commonly cited O_2_/˙O_2_^−^ potential of approximately −0.33 eV, suggesting that direct superoxide formation may be thermodynamically limited. However, these electrons can reduce dissolved oxygen through the two-electron pathway to form hydrogen peroxide, since the O_2_/H_2_O_2_ reduction potential is approximately +0.68 eV; the resulting H_2_O_2_ may subsequently generate additional hydroxyl radicals. Therefore, CIP degradation is expected to proceed mainly through direct oxidation by photogenerated holes and attack by hydroxyl radicals, with oxygen-derived species providing a secondary contribution.

## 5 Conclusion

A hierarchical flower-like Ag_3_PO_4_/CuInS_2_ heterostructure was successfully prepared through a combined solvothermal–hydrothermal route and evaluated for the visible-light-driven degradation of ciprofloxacin. Structural and surface analyses confirmed the coexistence and interfacial integration of cubic Ag_3_PO_4_ and chalcopyrite CuInS_2_, while electron microscopy revealed the close association of faceted Ag_3_PO_4_ particles with rough, flower-like CuInS_2_ aggregates. Coupling the two semiconductors increased the measured surface area, pore accessibility and visible-light response of the composite. The reduced electrochemical impedance and pronounced photoluminescence quenching further indicated improved interfacial charge migration and suppression of rapid electron–hole recombination. The Ag_3_PO_4_/CuInS_2_ heterostructure achieved 96.13% ciprofloxacin degradation after 60 min of visible-light irradiation, outperforming pristine Ag_3_PO_4_ and CuInS_2_, which attained 44.30% and 58.87% degradation, respectively. Its apparent pseudo-first-order rate constant of 0.03561 min^−1^ was approximately 5.3 times that of Ag_3_PO_4_ and 3.76 times that of CuInS_2_, although Ag_3_PO_4_/CuInS_2_ exhibited high photocatalytic activity towards CIP in laboratory-prepared solutions, its performance in real wastewater has not been established. Ciprofloxacin degradation was favoured near pH 6.6, where its zwitterionic form could interact effectively with the negatively charged catalyst surface. Increasing pollutant concentration reduced degradation because of competition for active sites, consumption of reactive species, accumulation of intermediates and attenuation of incident light. The scavenger experiments established that photogenerated holes and hydroxyl radicals were the major oxidative species, while superoxide-related species played a smaller role. A direct Z-scheme-like pathway was proposed in which interfacial recombination removed weak charge carriers while preserving strongly oxidizing holes in the Ag_3_PO_4_ valence band and reducing electrons in the CuInS_2_ conduction band. LC-MS results suggested that ciprofloxacin transformation proceeded through oxidation of the piperazine group, *N*-dealkylation, ring opening, deamination, defluorination, decarboxylation and progressive disruption of the quinolone framework. The composite retained 89.45% degradation after four cycles, corresponding to approximately 93.1% of its initial activity. Overall, the Ag_3_PO_4_/CuInS_2_ heterostructure shows promise for visible-light treatment of ciprofloxacin-contaminated water. Although the present study focused on ciprofloxacin degradation, the enhanced visible-light absorption and improved charge separation of the Ag_3_PO_4_/CuInS_2_ heterostructure suggest its potential applicability to other pharmaceutical contaminants, including tetracycline, sulfamethoxazole, amoxicillin, diclofenac, and carbamazepine. The involvement of photogenerated holes (h^+^) and hydroxyl radicals (˙OH) may facilitate the oxidative transformation of diverse pharmaceutical compounds. However, degradation efficiency is expected to vary depending on pollutant molecular structure, surface affinity, and susceptibility to oxidation. Future investigations should therefore evaluate the photocatalyst against a broader range of pharmaceuticals and explore compositional optimization and surface modifications to enhance its versatility and treatment efficiency.

## Conflicts of interest

The authors declare that they have no known competing financial interests or personal relationships that could have appeared to influence the work reported in this paper.

## Supplementary Material

RA-OLF-D6RA09598D-s001

## Data Availability

The data used in this study are mostly reported in the manuscript, and any additional data supporting the findings are available as supplementary information (SI). Supplementary information is available. See DOI: https://doi.org/10.1039/d6ra09598d.
