# Supplementary material for: Visible-light degradation of ciprofloxacin by hierarchical Ag3PO4/CuInS2 heterostructures: interfacial charge transfer, transformation pathways, and ecological risk context
Source: RSC Adv. 2026 Nov 12. Online ahead of print. doi: 10.1039/d6ra09598d (PMC13757426; doi:10.1039/d6ra09598d)
Supplement: Supplementary file 1 [file RA-OLF-D6RA09598D-s001.pdf]

Supplementary Information (SI) for RSC Advances.
This journal is © The Royal Society of Chemistry

Visible-light degradation of ciprofloxacin by hierarchical $Ag_3PO_4/CuInS_2$ heterostructures: Interfacial charge transfer, transformation pathways, and ecological risk assessment

Adewumi O. Oluwole[a], Nokwazi C. Mahlangu[a], Phephile Ngwenya[a], Shepherd M. Tichapondwa[a], Michael O. Daramolaa, Samuel A. Iwarerea*

[a] Department of Chemical Engineering, Faculty of Engineering, Built Environment and Information Technology, University of Pretoria, Hatfield, Pretoria 0002, South Africa

*Corresponding author. Tel: +27-12-420-3092. E-mail: omayor8@gmail.com (Adewumi Oluwole) and samuel.iwarere@up.ac.za (Samuel Iwarere)

Supplementary Information

Text SI: Occurrence of ciprofloxacin in global surface water matrices

The compiled occurrence data (**Fig. S1**) indicate that CIP is now pervasive in aquatic systems worldwide, with reported concentrations spanning more than five orders of magnitude. This variability is primarily governed by regional antibiotic consumption patterns, the efficiency of wastewater treatment technologies, the intensity of pharmaceutical manufacturing activities and the overall magnitude of anthropogenic pressures. In Europe, North America and Oceania, CIP concentrations in surface waters are typically within the ng/L to low μg/L range, a pattern that is consistent with comparatively advanced wastewater treatment infrastructure, more stringent environmental regulatory regimes and a reduced incidence of direct discharge of untreated effluents. Representative surface water concentrations include 0.005–0.192 μg/L in Chinese rivers (Luo et al., 2011), 0.001–0.160 μg/L in Sweden, 0.005–0.018 μg/L in Switzerland, 0.001–0.031 μg/L in the United States and 0.001–0.017 μg/L in Canada, with values generally below 0.01 μg/L in Australia and New Zealand [1-6]. Despite these comparatively low background levels, pronounced local enrichment is frequently observed downstream of wastewater treatment plants, demonstrating that conventional wastewater treatment processes are insufficient to completely eliminate fluoroquinolone antibiotics. This limitation is largely attributable to their physicochemical

properties, including high environmental persistence, strong sorption to suspended particulate matter and limited susceptibility to microbial biodegradation [7]. Moderate CIP concentrations have also been documented in South American surface waters, for example in Brazil, Argentina, and Peru, which can be associated with rapid urban expansion and increasing wastewater loads but generally remain below the highest values reported for some developing regions [8, 9]. These observations demonstrate that while CIP contamination is now a globally pervasive phenomenon, the magnitude of contamination is strongly modulated by the performance of regional wastewater management systems and the rigor of environmental regulatory frameworks.

In contrast, substantially elevated CIP concentrations have been reported across Africa and parts of Asia, regions where insufficient wastewater treatment capacity, direct discharge of untreated municipal effluents, intensive antibiotic usage, and emissions from pharmaceutical manufacturing collectively result in pronounced environmental contamination. In South Africa, riverine CIP concentrations ranged from 0.03 – 2.35 μg/L, whereas surface waters in Nigeria reached 17.5 μg/L and pharmaceutical effluent discharge zones exhibited levels up to 12.8 μg/L [10-12]. Similarly, surface waters in Kenya, Ethiopia, and Egypt contained concentrations exceeding 2 μg/L at specific locations, while exceptionally high underscoring severe contamination linked to inadequate wastewater infrastructure [4, 13, 14]. Asian aquatic systems exhibited exorbitantly pronounced contamination, particularly in India, where concentrations reached up to 31 000 μg/L in the Godawari River system, reflecting the impacts of pharmaceutical manufacturing clusters, hospital effluents, and untreated domestic sewage [15, 16]. Analogous contamination patterns were reported for Pakistan, Vietnam, and Bangladesh, although concentrations in Chinese rivers were generally below 0.32 μg/L despite their ubiquitous detection [1, 17-19]. These global occurrence data indicate that CIP is now among the most widely distributed pharmaceutical contaminants in aquatic environments, with contamination hotspots predominantly located in low- and middle-income countries where limited wastewater treatment capacity and largely uncontrolled antibiotic discharges sustain continuous environmental loading. This pervasive occurrence is of particular concern because fluoroquinolones can elicit ecotoxicological effects on aquatic microorganisms even at sub-μg/L

concentrations and may contribute to the selection and propagation of antimicrobial resistance genes in receiving water bodies[7, 16].

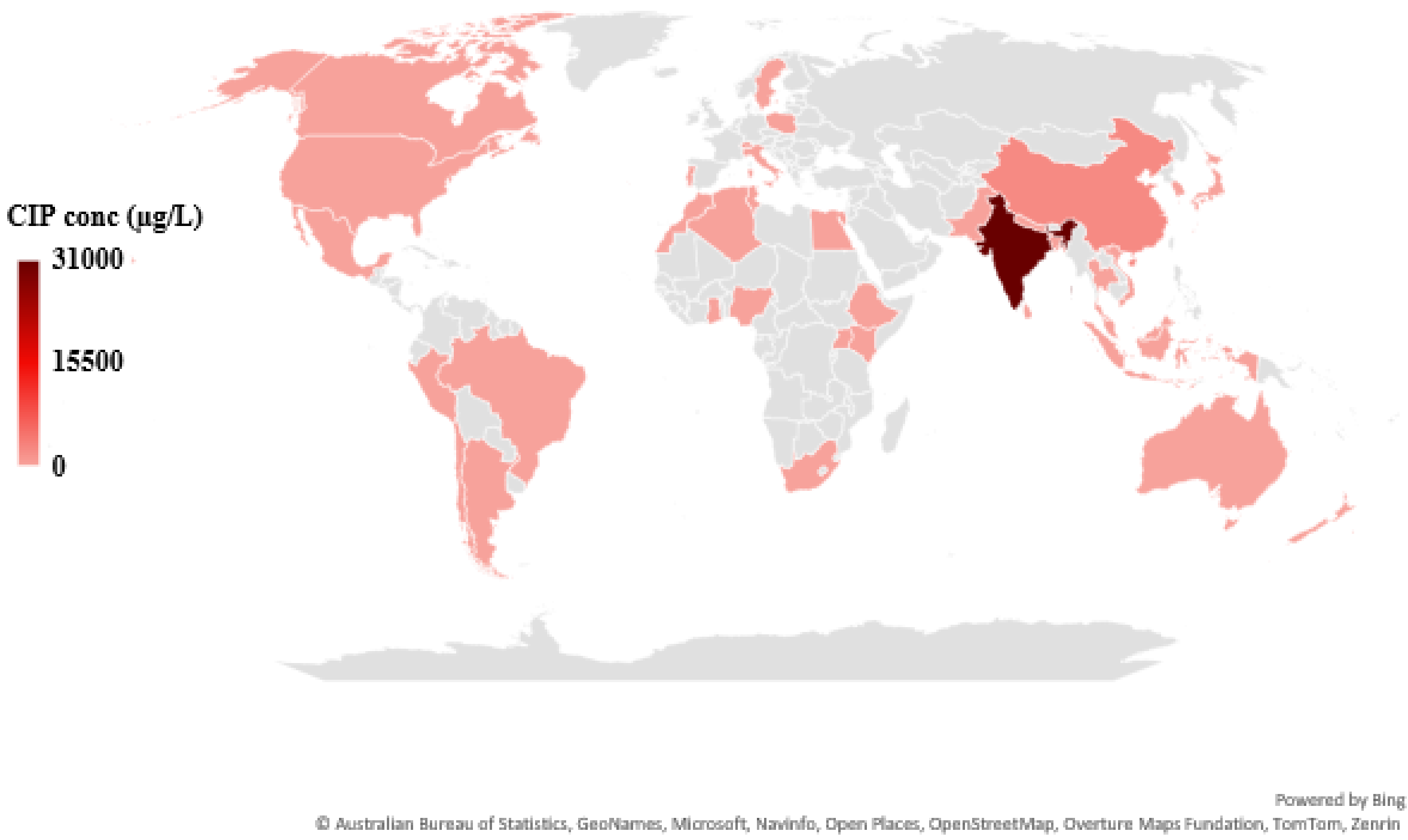

Fig. S1: Global measured environmental concentrations of ciprofloxacin.

Text S2: Characterization instrumentation details

The crystal phase of the prepared samples was characterized by X-ray diffraction (XRD) using a PANalytical X'Pert Pro powder X-ray diffractometer with Cu-Kα radiation (λ = 0.15406 Å) in θ–θ configuration at an acceleration voltage and current of 40 kV and 15 mA. The analysis was performed over a diffraction angle range of 2θ range of 5–80° with a step size of 0.02° and a scanning rate of 3° min^{-1}. The surface functional groups and vibrational modes, providing insight into the bonding environment and structural integrity of the composite samples were examined by PerkinElmer Spectrum One spectrometer and WITec alpha300R Confocal Raman Microscope. The morphology and elemental composition of the materials were observed using a field-emission scanning electron microscopy–energy dispersive X-ray spectrometry (SEM-EDS; JEOL Scanning Microscope JSM-6400). X-ray photoelectron spectroscopy (XPS) characterization using a Kratos Axis supra$^+$, coupled with a monochromatic Al Ka (hv = 1486.6 eV) and running at 150 W (XPS), was used to determine the chemical composition, oxidation state of the elements, and formation of heterostructure composites within the Ag_3PO_4/$CuInS_2$. The surface area, average pore volume, and pore-size distribution of the synthesized composites were measured using a Brunauer–Emmett–Teller analyzer (Micromeritics Tristar II 3020, Version 3.02) after overnight degassing at 100 °C prior to analysis. Optical properties were characterized using UV diffuse reflectance spectroscopy (DRS) with a UV-vis spectrophotometer (Agilent Technologies Cary 60 UV-vis, Malaysia), where $BaSO_4$ was used as a reference. Photoluminescence (PL) spectroscopy was performed using a Shimadzu RF-5301 spectrofluorophotometer with an excitation wavelength of 250 nm while the electrochemical impedance spectroscopy measurements were done using an Autolab Potentiostat workstation (PGSTAT204, Netherlands) to investigate the interfacial charge-transfer properties, semiconductor-type behaviour, and photoelectrochemical response of the synthesized

heterostructure composites. After the photodegradation of CIP using the synthesized composite materials, the remaining comcentration in the solution were measured using a UV-vis spectrophotometer (Biochrom, Cambridge, UK), while degradation product analysis was carried out using the Liquid Chromatography-Mass Spectrometry (LC-MS) XEVO G2-XS QTof.

Text S3. Crystalline size calculation for the composite materials

$$D = \frac{K\lambda}{\beta COS\theta} \qquad (1)$$

Where D=crystallite size, K=0.9, λ=0.15406 nm for Cu Kα, β=FWHM in radians and θ=Bragg angle

Table S1. XPS peak-fitting parameters, including binding energies (eV), full width at half maximum (FWHM, eV), and integrated peak areas for pristine Ag_3PO_4, $CuInS_2$, and $Ag_3PO_4/CuInS_2$ heterostructure, estimated from survey spectra using Gaussian peak fitting.

Ag_3PO_4

Peak	Binding energy (eV)	FWHM (eV)	Peak Area
Ag $3d_{5/2}$	367,71	2,23	495,132
Ag $3d_{3/2}$	373,76	2,32	364,514
P 2p	133,03	2,99	37,801
O 1s	531,12	3,50	265,834

$CuInS_2$

Peak	Binding energy (eV)	FWHM (eV)	Peak Area
Cu $2p_{3/2}$	932,42	2,7	278474
In $3d_{5/2}$	445,09	2,66	844293
In $3d_{3/2}$	452,65	2,65	572153
S 2p	162,18	2,97	132422
O 1s	531,97	2.97	208825

$Ag_3PO_4/CuInS_2$

Peak	Binding energy (eV)	FWHM (eV)	Peak Area
Ag 3d5/2	367,96	2,34	583662
Ag 3d3/2	374,01	2,38	413864
P 2p	133,15	2,89	41377
O 1s	531,25	3,38	283919
Cu $2p_{3/2}$	930,02	2,57	278474
In 3d5/2	445,07	2,52	47450
In 3d3/2	452,63	2,48	31841
S 2p	160,02	2,75	122292

Table S2: Comparison of $Ag_3PO_4/CuInS_2$ heterostructure composites with other material in CIP removal

Materials	Methodological approaches	Conditions	Dosage (mg/L)	Removal efficiency (%)	References

$g\text{-}C_3N_4/Gd_2O_3$	Facile ultrasonication-assisted	125 W high-pressure mercury lamp, 100 mL, 20 mg/L	0.1 g	97 in 2 h	30
$g\text{-}C_3N_4/Sm_2O_3$	Simple in situ hydrothermal method	125 W high-pressure mercury lamp, 100 mL, 30 mg/L	0.5 g	90.6 in 2	31
Ag_3PO_4–WO_3)/coffee bean biochar	Hydrothermal method	125 W high-pressure mercury lamp, 100 mL 50 mg/L,	0.1 g	99 in 1 h	32
CoF/PmAP	Hydrothermal method	125 W high-pressure mercury lamp, 150 mL 50 mg/L,	0.1 g	99 in 1 h	33
CdS-Bi_2MoO_6	Hydrothermal method	300 W xenon lamp, 100 mL, 20 mg/L	0.3	87 % in 1 h	34
GO/ZnO/Ag	Hydrothermal method	xenon lamp, 50 mL, 20 mg/L	15	82.13 in 3 h	35

g-C_3N_4/MoS_2	One-pot synthesis method	8 × 8 W LED lamp, 20 mg/L	0.5	90 in 1 h	[36]
Ag_3PO_4/$CuInS_2$	Solvo-hydrothermal method	72 W fluorescent daylight lamp, 500 mL, 7 mg/L	0.05	96.13 in 1h	This study

Table S3: Summary of the degradation and kinetic data

Materials	Degradation (%)	Rate Constant □ (min^{-1})	R^2
Photolysis	8.75	0.00112	0.9865
Ag_3PO_4	44.30	0.00678	0.9643
$CuInS_2$	58.87	0.00948	0.9405
$Ag_3PO_4/CuInS_2$	96.13	0.03561	0.9104

Fig. S2. XRD patterns of $Ag_3PO_4/CuInS_2$ heterostructure before and after four consecutive photocatalytic degradation cycles.

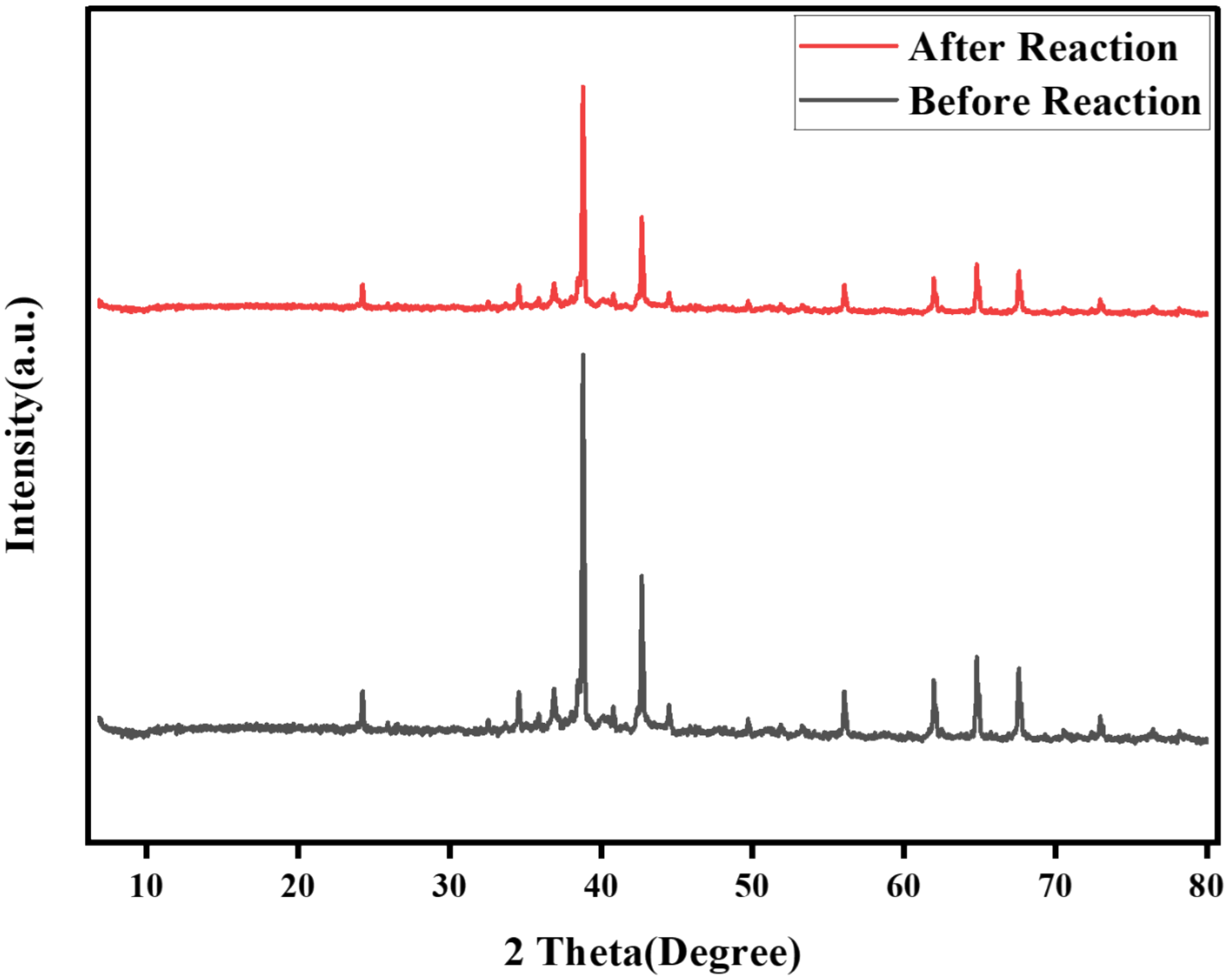

Fig. S3: MS chromatogram of CIP and the corresponding mass spectra after 60 min of irradiation by $Ag_3PO_4/CuInS_2$ heterostructure composites

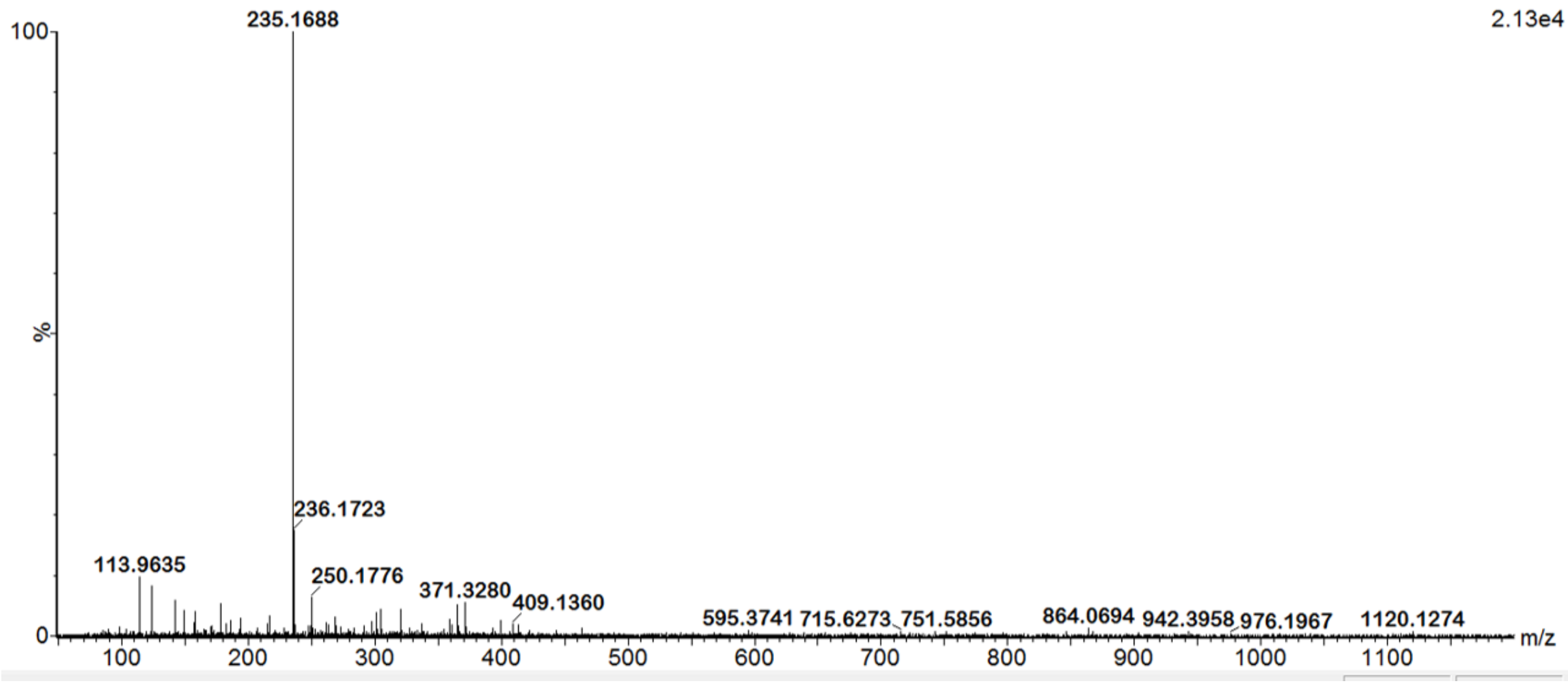

Fig. S4. TOC removal rate for Ag_3PO_4, $CuIn_2$ and $Ag_3PO_4/CuInS_2$ heterostructure composites

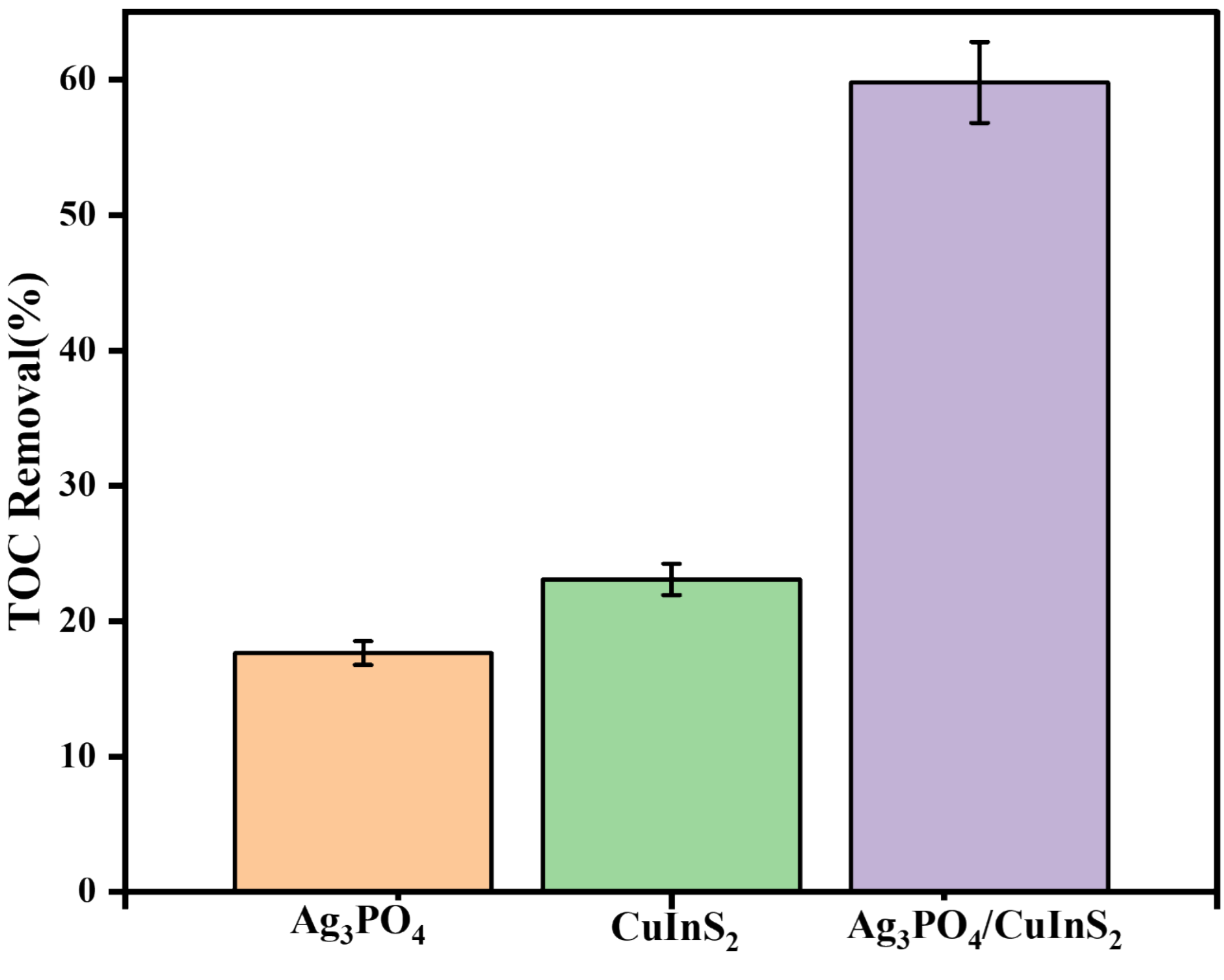

Table S4: Summary of studies reporting on the ecotoxicity of CIP on aquatic biomarkers.

Organism (Scientific name)	Exposure time (h)	Exposure concentration (mg/L)	$E(L)C_{50}$ NOEC (mg/L)	Effect on organism	Reference
Microcystis aeruginosa	120	0.0079–1.96	0.0079	Strong inhibition of growth; most sensitive aquatic organism tested	[20]
Pseudokirchneriella subcapitata	72	0.5–25	1.10	Growth inhibition and reduced photosynthetic activity	[20]

Pseudokirchneriella subcapitata	72	0.5–100	2.0	Reduced algal growth rate; chlorophyll inhibition	21
Lemna minor	168	0.01–5	0.053	Significant inhibition of frond growth and biomass production	20
Lemna minor	168	0.01–5	0.24	Reduced frond number and growth rate	21
Spirodela polyrhiza	168	0.05–2	0.28	Reduced frond area and biomass accumulation	22
Daphnia magna	48	□10	10	No significant immobilization or mortality observed	20
Daphnia magna	48	1–100	5	Immobilization and reduced swimming activity	22
Daphnia magna	504	0.056–28	0.056	Reduced reproduction and offspring production after chronic exposure	23
Daphnia magna	48	N/A	N/A	Oxidative stress, GST induction, altered cholinesterase activity, impaired swimming behaviour and smaller neonates	24
Ceriodaphnia dubia	72	1–50	3 – 23	Mortality and reproductive impairment	25

Daphnia magna	48	5–80	14 – 73	Acute mortality following quinolone exposure (including ciprofloxacin)	25
Gambusia holbrooki	96	Up to 100	> 100	No acute mortality observed; low sensitivity to ciprofloxacin	21
Pimephales promelas	168	□10	10	No mortality; slight increase in dry weight reported	20
Microcystis aeruginosa	120	0.0079–1.96	0.0079	Severe inhibition of growth and photosynthetic activity; highly sensitive species	20
Anabaena flos-aquae	72	0.001–0.05	0.0102	Growth inhibition and reduced cell yield	26
Pseudokirchneriella subcapitata	72	1.1–22.7	1.10	Growth inhibition and reduced biomass production	20
Chlorella vulgaris	72	0–20	9.23	Significant inhibition of algal growth	27
Chlorella vulgaris	72	<20	< 20	No observable inhibition below 20 mg/L	28
Cylindrotheca closterium	96–120	5–100	55.43	Growth inhibition	28
Nitzschia ramosissima	96–120	5–100	72.12	Reduced cell proliferation	28

Daphnia magna	48	□10	10	No significant mortality at concentrations up to 10 mg/L	20
Daphnia magna	48	14–73	14	Acute immobilisation and mortality	25
Daphnia magna	504	4–28	4	Reduced reproduction, impaired growth and delayed maturation	25
Ceriodaphnia dubia	72	3–23	3	Mortality and reduced survival; more sensitive than D. magna	25
Ceriodaphnia dubia	168–504	4–28	4	Decreased fecundity and impaired life-history traits	25
Daphnia magna	48–504	0.0001–0.1	N/A	Oxidative stress, cholinesterase inhibition, altered catalase and GST activity, behavioural impairment, reduced energy reserves and production of smaller neonates	24
Pimephales promelas	168	□10	10	No significant mortality; slight increase in larval dry weight reported	20
Danio rerio	48	>2	2	Low acute toxicity; limited embryo lethality	28
Microcystis panniformis	96		0.01358	Growth rate inhibition	29

References

1. Y. Luo, L. Xu, M. Rysz, Y. Wang, H. Zhang and P. J. Alvarez, Environmental science & technology, 2011, 45, 1827–1833.
2. R. H. Lindberg, K. Björklund, P. Rendahl, M. I. Johansson, M. Tysklind and B. A. Andersson, Water research, 2007, 41, 613–619.
3. E. M. Golet, A. C. Alder and W. Giger, Environmental science & technology, 2002, 36, 3645–3651.
4. J. L. Wilkinson, A. B. Boxall, D. W. Kolpin, K. M. Leung, R. W. Lai, C. Galbán-Malagón, A. D. Adell, J. Mondon, M. Metian and R. A. Marchant, Proceedings of the national academy of sciences, 2022, 119, e2113947119.
5. X.-S. Miao, F. Bishay, M. Chen and C. D. Metcalfe, Environmental science & technology, 2004, 38, 3533–3541.
6. I. Pattis, L. Weaver, S. Burgess, J. E. Ussher and K. Dyet, Antibiotics, 2022, 11, 778.
7. K. Kümmerer, Journal of environmental management, 2009, 90, 2354–2366.
8. J. I. Nieto-Juárez, R. A. Torres-Palma, A. M. Botero-Coy and F. Hernández, Environment international, 2021, 155, 106674.
9. C. M. Teglia, F. A. Perez, N. Michlig, M. R. Repetti, H. C. Goicoechea and M. J. Culzoni, Environmental Toxicology and Chemistry, 2019, 38, 2305–2313.
10. F. O. Agunbiade and B. Moodley, Environmental Toxicology and Chemistry, 2016, 35, 36–46.
11. E. O. Fakorede, F. O. Ajibade, J. R. Adewumi and J. O. Ighalo, Regional Studies in Marine Science, 2026, 104842.
12. L. M. Madikizela, N. T. Tavengwa and L. Chimuka, Journal of environmental management, 2017, 193, 211–220.
13. M. S. Fouad, E. F. Mustafa, M. S. Hellal and M. A. Mwaheb, Scientific Reports, 2024, 14, 18849.
14. P. Kairigo, E. Ngumba, L.-R. Sundberg, A. Gachanja and T. Tuhkanen, Water, 2020, 12, 1376.
15. J. Fick, H. Söderström, R. H. Lindberg, C. Phan, M. Tysklind and D. J. Larsson, Environmental toxicology and chemistry, 2009, 28, 2522–2527.
16. D. J. Larsson, C. De Pedro and N. Paxeus, Journal of hazardous materials, 2007, 148, 751–755.
17. G. Zhao, Q. Lu, S. Ye, H. Yuan, X. Ding and J. Wang, Marine pollution bulletin, 2016, 108, 268–274.
18. V. Thamlikitkul, S. Tiengrim, N. Thamthaweechok, P. Buranapakdee and W. Chiemchaisri, International Journal of Environmental Research and Public Health, 2019, 16, 3753.
19. M. A. Islam, B. Das, S. B. Quraishi, R. Khan, K. Naher, S. M. Hossain, S. Karmaker, S. A. Latif and M. B. Hossen, Marine Pollution Bulletin, 2020, 160, 111649.

20. A. A. Robinson, J. B. Belden and M. J. Lydy, Environmental toxicology and Chemistry, 2005, 24, 423–430.
21. N. Martins, R. Pereira, N. Abrantes, J. Pereira, F. Gonçalves and C. Marques, Ecotoxicology, 2012, 21, 1167–1176.
22. E. Pietropoli, R. Zgheib, M. Selmo, G. Melotto, R. M. Lopparelli, L. Lucatello, M. Pauletto and M. De Liguoro, Toxics, 2025, 13, 979.
23. D. Eluk, O. Nagel, A. Gagneten, U. Reno and R. Althaus, Water Environment Research, 2021, 93, 2914–2930.
24. R. Dionisio, D. Daniel, G. D. de Alkimin and B. Nunes, Environmental toxicology and pharmacology, 2020, 74, 103295.
25. S. V. Kergaravat, N. Romero, L. Regaldo, G. R. Castro, S. R. Hernández and A. M. Gagneten, Microchemical Journal, 2021, 162, 105832.
26. I. Ebert, J. Bachmann, U. Kühnen, A. Küster, C. Kussatz, D. Maletzki and C. Schlüter, Environmental toxicology and chemistry, 2011, 30, 2786–2792.
27. J.-Q. Xiong, M. B. Kurade, J. R. Kim, H.-S. Roh and B.-H. Jeon, Journal of hazardous materials, 2017, 323, 212–219.
28. Q. Yang, Y. Gao, J. Ke, P. L. Show, Y. Ge, Y. Liu, R. Guo and J. Chen, Bioengineered, 2021, 12, 7376–7416.
29. F. C. R. Azevedo, I. C. D. Vaz, F. A. R. Barbosa and S. M. S. Magalhães, Brazilian Journal of Pharmaceutical Sciences, 2019, 55, e17661.
30. P. P. Mishra, D. Nanda, S. Sahoo, N. Das, S. Manna and A. K. Behera, Journal of Industrial and Engineering Chemistry, 2025.
31. P. P. Mishra, C. Mohanty, A. K. Behera, R. Das and N. Das, Journal of Rare Earths, 2025.
32. D. Nanda, C. Mohanty, A. Priyadarsini, N. Das, M. Misra, A. K. Mohanty and A. K. Behera, RSC advances, 2025, 15, 26787–26799.
33. C. Mohanty, A. Samal, A. K. Behera and N. Das, Journal of Water Process Engineering, 2024, 65, 105821.
34. C. Dai, L. He, M. Zhao, F. Teng, Y. Sang, J. Li, Y. Li, Y. Zheng and M. Wu, ChemistrySelect, 2024, 9, e202303076.
35. H. Chi, P. Cao, Q. Shi, C. Song, Y. Lv and T. Peng, Nanomaterials, 2025, 15, 383.
36. P. Pal, Z.-F. Lin and R.-A. Doong, Chemical Engineering Journal, 2024, 502, 157884.
